# Supplementary material for: Simultaneously ultrafast and robust two-dimensional flash memory devices based on phase-engineered edge contacts
Source: Nat Commun. 2023 Sep 13;14:5662. doi: 10.1038/s41467-023-41363-x (PMC10499832; doi:10.1038/s41467-023-41363-x)
Supplement: Supplementary file 1 — Supplementary Information [file 41467_2023_41363_MOESM1_ESM.pdf]

Supplementary Information

Simultaneously Ultrafast and Robust Two-dimensional Flash Memory Devices Based on Phase Engineered Edge Contacts

Jun Yu[1], Han Wang[1], Fuwei Zhuge[1*], Zirui Chen[2], Man Hu[1], Xiang Xu[1], Yuhui He[2], Ying Ma[1*], Xiangshui Miao[2], Tianyou Zhai[1*]

1. State Key Laboratory of Materials Processing and Die and Mould Technology, School of Material Science and Engineering, Huazhong University of Science and Technology, Wuhan 430074, China.

2. Wuhan National Laboratory for Optoelectronics; School of Optical and Electronic Information and Hubei Key Laboratory of Advanced Memories, Huazhong University of Science and Technology, Wuhan 430074, China

Corresponding to: zhugefw@hust.edu.cn, yingma@hust.edu.cn, zhaity@hust.edu.cn

Table of content

Supplementary Note 1. Fabrication of flash memory cell.

Supplementary Note 2. Characterization of the heterostructure

Supplementary Note 3. Li intercalation induced phase transition.

Supplementary Note 4. Hysteresis in edge contacted memory cell

Supplementary Note 5. Waveform verification

Supplementary Note 6. Tunneling carrier and barrier in memory operation

Supplementary Note 7. Speed estimation of an ideal flash memory

Supplementary Note 8. Estimation of gate coupling ratio

Supplementary Note 9. Validation of the role of edge contact using paired memory cell

Supplementary Note 10. P/E heat map for additional edge contacted memory cells.

Supplementary Note 11. Evaluation of tunneling current density in differently configured heterostructure

Supplementary Note 12. Effect of float gate choice to memory operation

Supplementary Note 13. Estimation of trap density in memory cells

Supplementary Note 14. Potential sources of trap states in device fabrication

Supplementary Note 15. Extraction of Schottky barrier at contact

Supplementary Note 16. Temperature accelerated retention measurement

Supplementary Note 17. Retention behavior during cycled endurance measurement

Supplementary Note 18. Comparison of endurance lifetime with edge and top contact configuration

Supplementary Note 19. Performance comparison to the state of art flash memory cells

Supplementary Note 20. Realization of 4-bit memory states

Supplementary Note 1. Fabrication of flash memory cell.

The fabrication of edge contacted memory cell follows the flowchart in Supplementary Figure 1, while the fabrication of paired memory cells that compares edge contact and top contact in the same heterostructure follows the flowchart in Supplementary Figure 2.

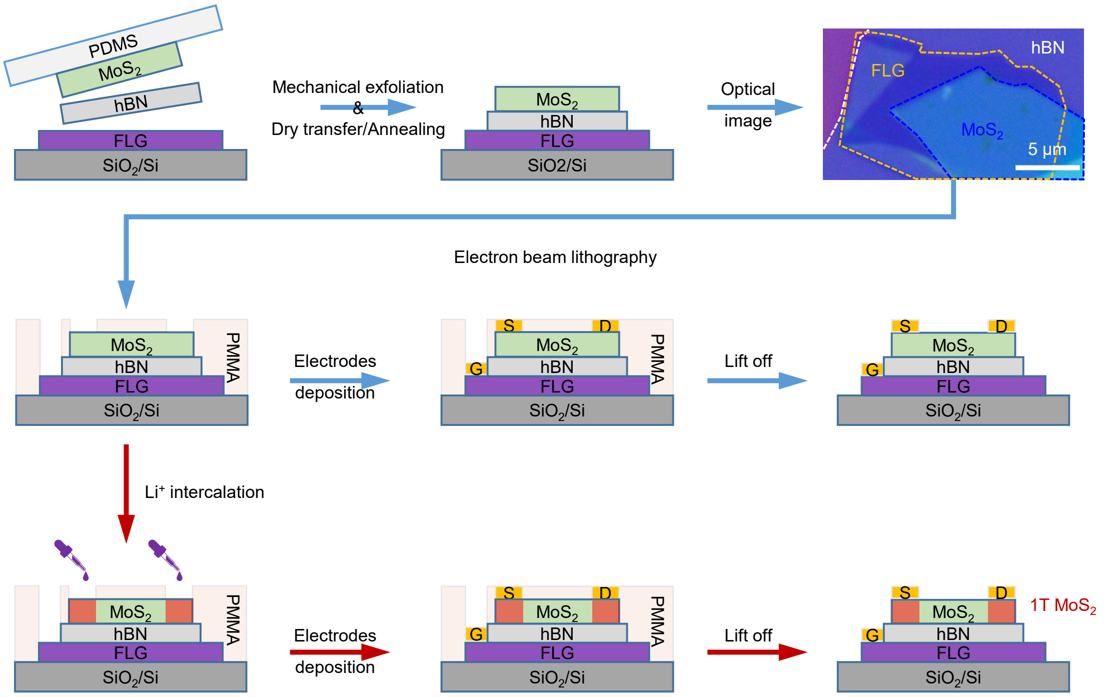

Supplementary Figure 1. Fabrication process for the MoS_2/hBN/FLG float gate transistor with traditional top contact or 1T-Li_xMoS_2 edge contact

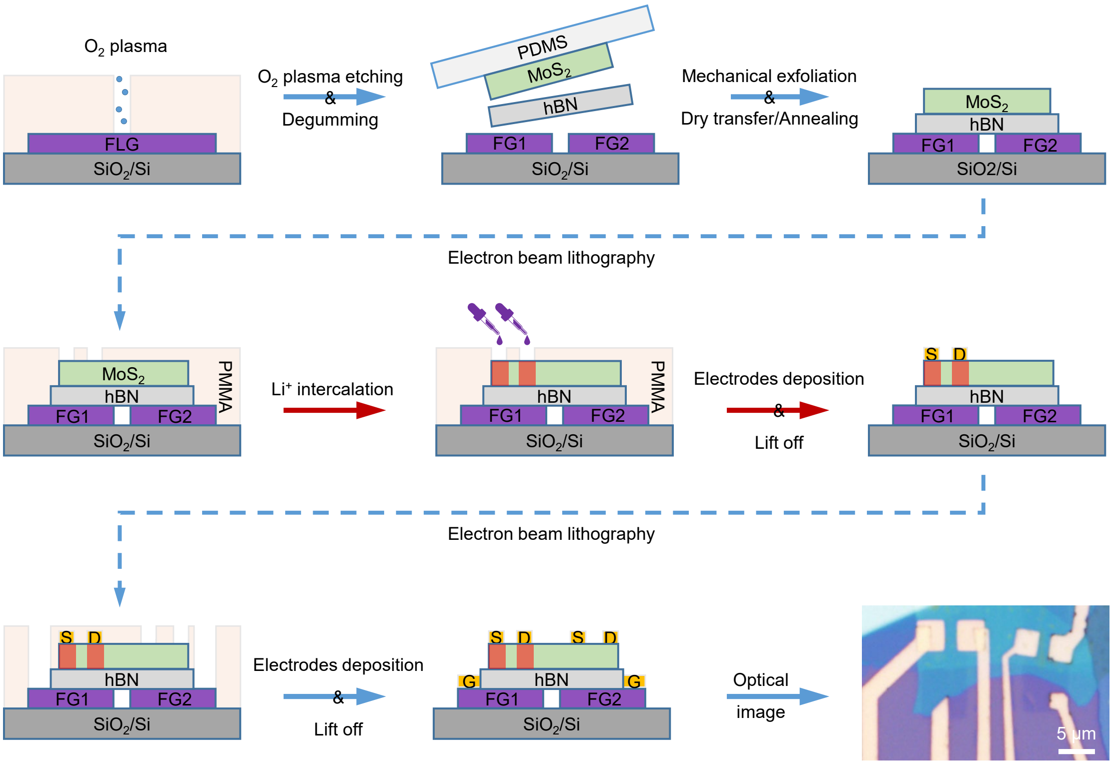

Supplementary Figure 2. Fabrication process for paired MoS_2/hBN/FLG float gate memory cells with distinct contact configuration.

Supplementary Note 2. Characterization of the heterostructure.

The heterostructure after stacking is measured using Raman spectra (Supplementary Figure 3), while the thickness of each layer is determined using AFM (Supplementary Figure 4). For devices reported in this work, the average thickness of MoS_2 and hBN is ~3-5 nm and 11-12 nm (Supplementary Figure 5 and 6).

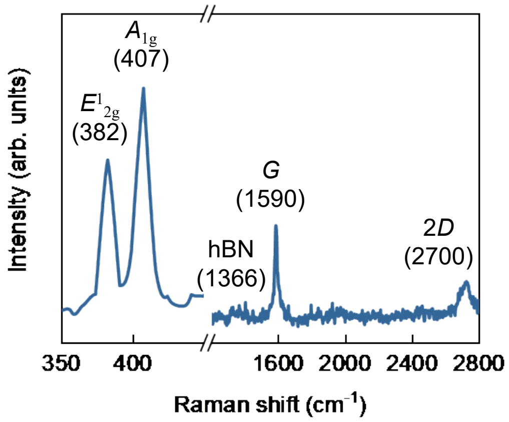

Supplementary Figure 3. Raman spectra of the FLG/hBN/MoS_2 heterostructure. The two typical Raman shifts of MoS_2 E^1_{2g} and A_{1g} are at 382 and 407 cm^{-1}, respectively. The hBN E_{2g} peak is at 1366 cm^{-1}, and the few-layer graphene G peak is at 1590 cm^{-1} and 2D peak at 2700 cm^{-1}.

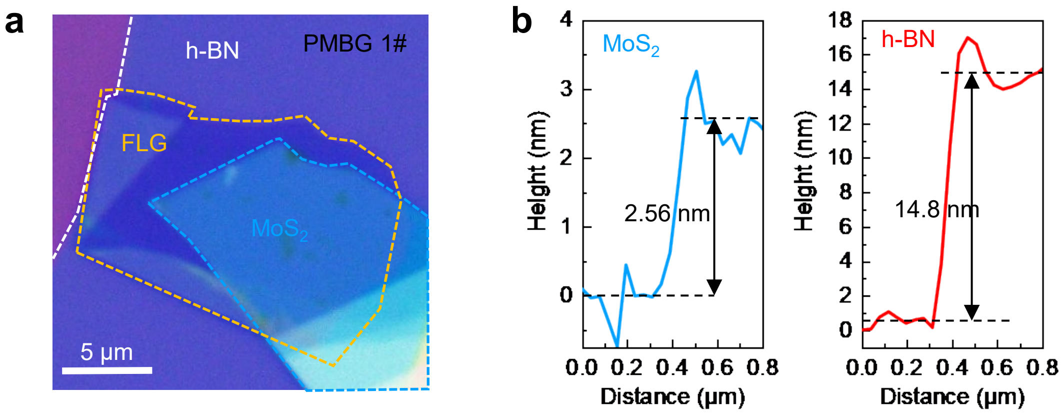

Supplementary Figure 4. Thickness of MoS_2 and hBN flakes in the vdW heterostructure. a, Optical microscope image of the MoS_2/hBN/FLG vdW heterostructure. The boundaries of MoS_2, hBN and FLG are marked by blue, white and orange dashed lines, respectively. The scale bar is 5 μm. **b,** The determined thickness of MoS_2 and hBN are 2.56 and 14.8 nm, respectively, according to step height measurement with atomic force microscopy.

Thickness of hBN and MoS_2 for top and edge contacted devices in main test.				
PMBG2#<br>hBN: 11.0 nm<br>MoS_2: 4.7 nm	PMBG3#<br>hBN: 9.7 nm<br>MoS_2: 4.7 nm	PMBG4#<br>hBN: 9.3 nm<br>MoS_2: 1.9 nm	PMBG5#<br>hBN: 9.7 nm<br>MoS_2: 7.2 nm	PMBG6#<br>hBN: 12.3 nm<br>MoS_2: 3.6 nm
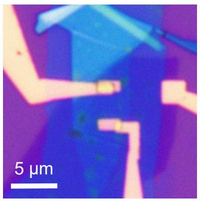	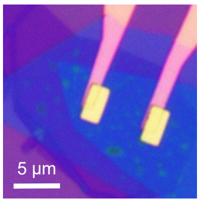	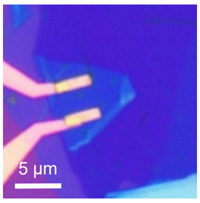	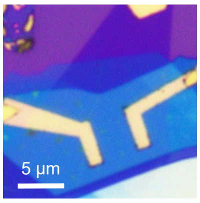	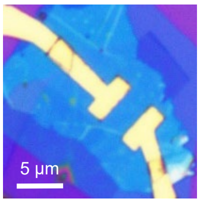
PMBG7#<br>hBN: 9.9nm<br>MoS_2: 1.8 nm	PMBG19#<br>hBN: 12.8 nm<br>MoS_2: 2.9 nm	MBG1#<br>hBN: 10.1 nm<br>MoS_2: 1.7 nm	MBG2#<br>hBN: 10.5 nm<br>MoS_2: 5.0 nm	MBG3#<br>hBN: 9.6 nm<br>MoS_2: 2.44 nm
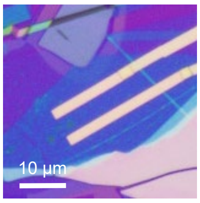	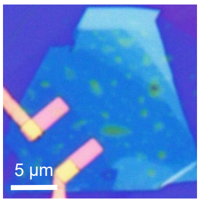	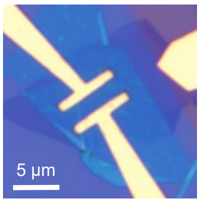	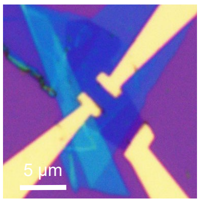	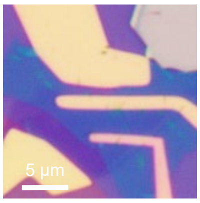

Supplementary Figure 5. Thickness of hBN and MoS_2 for different top (MBG) and edge (PMBG) contacted devices appeared in main text and their optical microscope images.

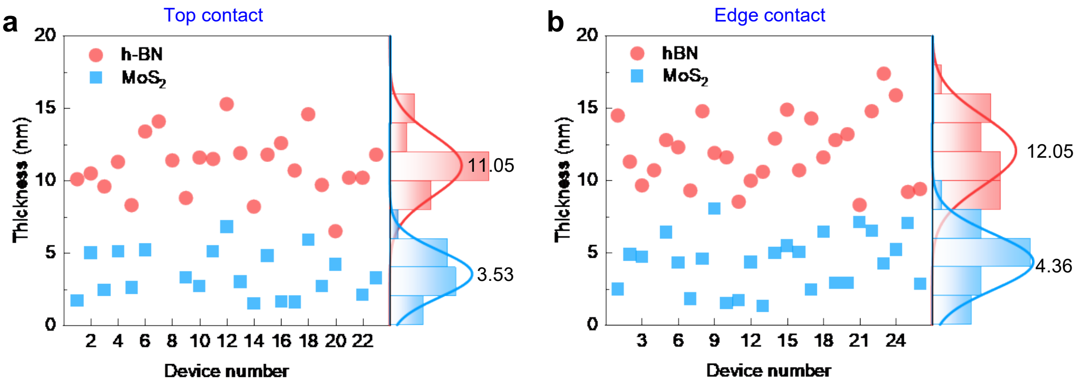

Supplementary Figure 6. Thickness statistics of hBN and MoS_2 in all fabricated flash memory devices displayed in Figure 2c and d. a, top contacted devices. **b**, edge contacted devices. Normal distribution is used to fit the thickness distribution in devices. For top contacted memory cells, the average thickness of hBN and MoS_2 is 11.05 nm and 3.53 nm, while for edge contacted memory cells, the value is 12.05 and 4.36 nm.

Supplementary Note 3. Li intercalation induced phase transition.

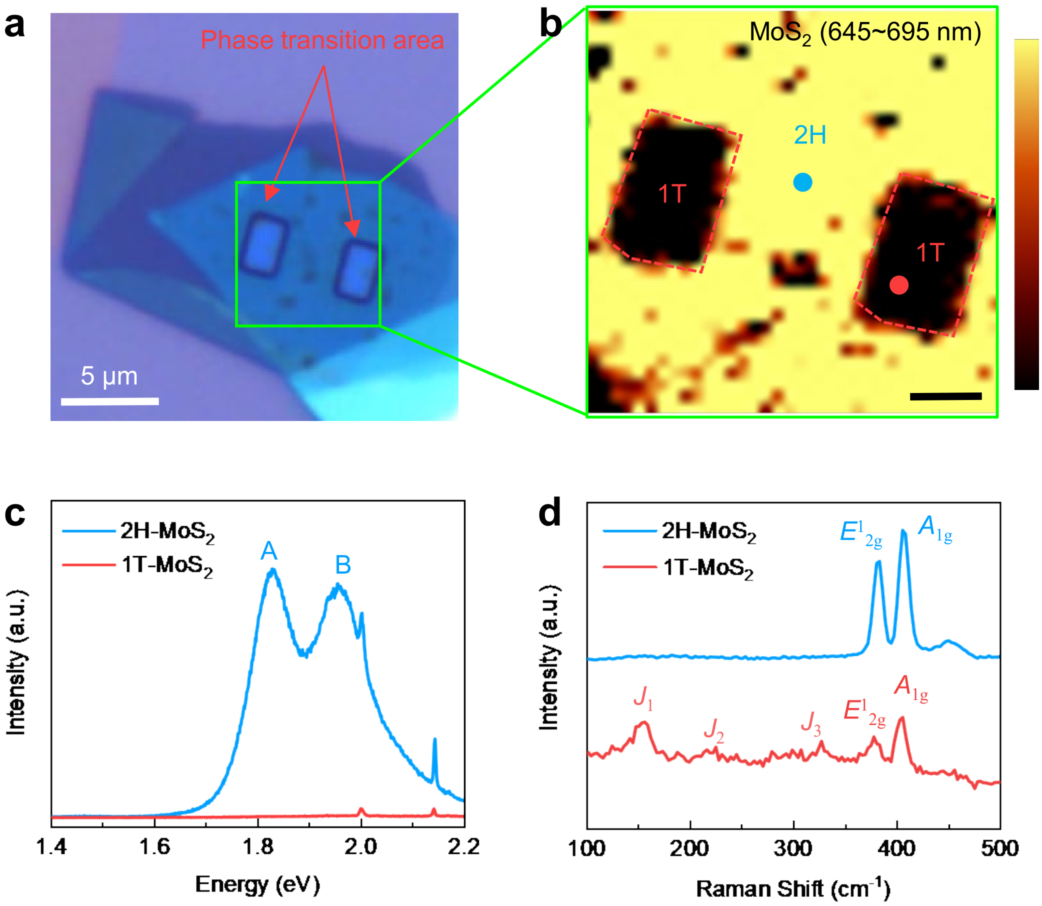

Supplementary Figure 7. Photoluminescence (PL) evaluation of patterned lithium intercalation in MoS_2 for 1T edge contact. a, Optical microscope image of the heterostructure after n-BuLi intercalation. The two rectangular windows were firstly patterned by EBL and then exposed to n-BuLi solution. **b,** A spatial mapping of PL intensity (645–695 nm) at chosen patterned area in **a**. **c, d** are respectively the PL and Raman spectra taken at selected spots in **b**. The extinction of PL intensity at ~1.8 and 1.95 eV and the appearance of additional Raman shift at 154 cm^{-1} (J_1), 224 cm^{-1} (J_2) and 327 cm^{-1} (J_3) indicated the transformation to metallic 1T-Li_xMoS_2.

Supplementary Note 4. Hysteresis in edge contacted memory cell

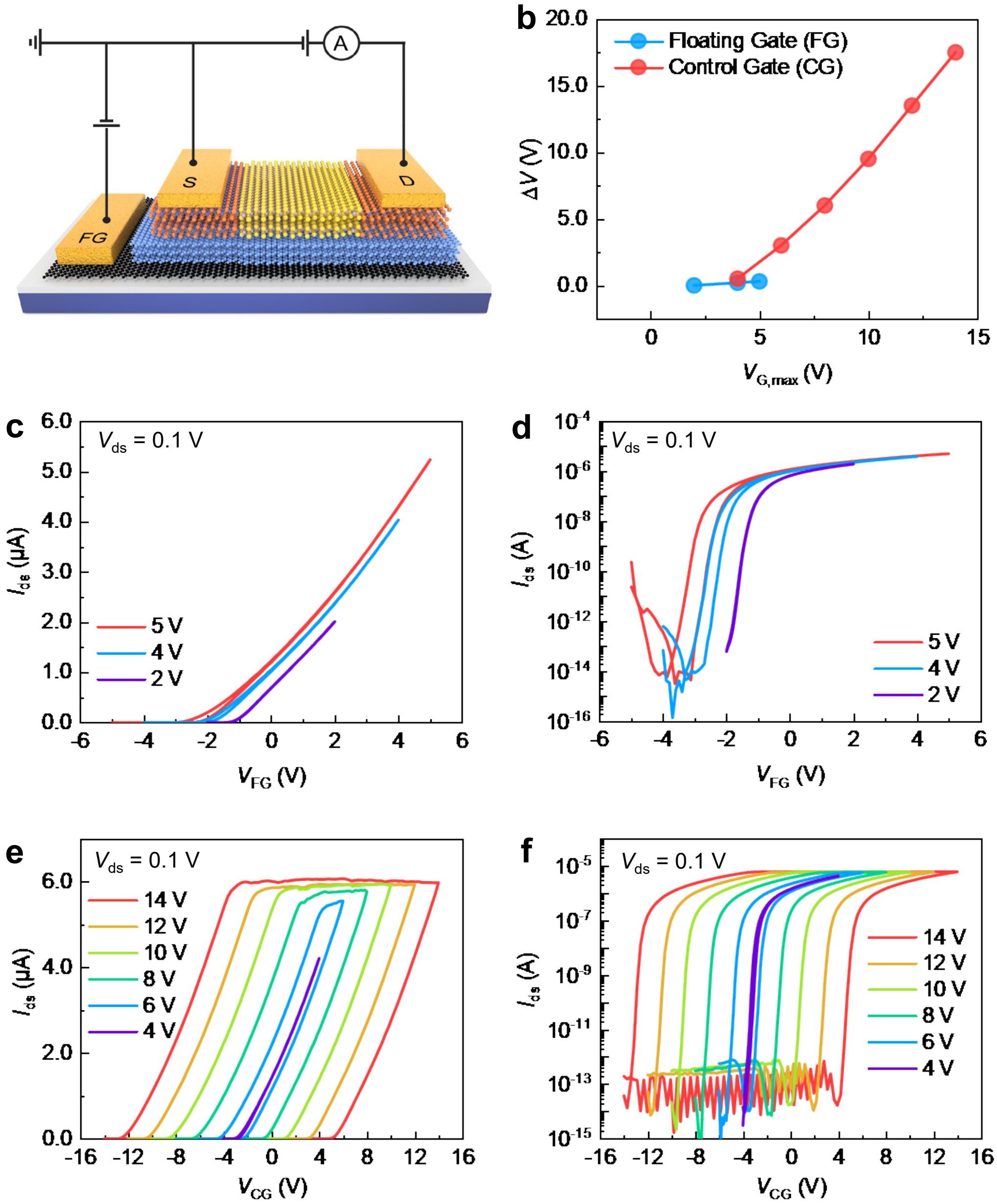

Supplementary Figure 8. Memory window of the edge contacted flash memory device. a, Structure diagram of the edge contacted flash memory device. **b,** The comparison of hysteresis window when sweeping V_{CG} and V_{FG}. Almost no hysteresis is observed when sweeping V_{FG}, while a large memory window can be obtained by sweeping V_{CG}, which confirms the origin of memory states by charge trapping in float gate rather than defect states in semiconductor or dielectric. The supporting transfer curves when dual-sweeping V_{FG} and V_{CG} in different ranges are displayed in **c** and **e**, respectively. **d** and **f** show the same results with current axis in logarithmic scale to indicate the high ON/OFF ratio of present memory cell.

Supplementary Note 5. Waveform verification

To probe the pulse waveforms directly applied to the devices, we had performed direct measurement of waveform by placing the passive probe of the microscope to the contact probe of Lakeshore probe station. **Supplementary Figure 9a** shows the adopted experimental setup, following Ref.[1]. In order to increase the reliability of the contact, the two probes are contacted to the metal indium (red dash line region), which resembles the case of connecting probe to back-Si control gate.

Through this method, a series of pulses with different amplitudes (±5 V, ±10 V, ±15 V and ±20 V) and different pulse widths (10 ns, 20 ns, 50 ns, 100 ns and 200 ns) are measured, in which the input impedance for oscilloscope is set at a high impedance of 1MΩ to resemble the case of signal coupling to high impedance gate terminal of the device. The observed voltage waveform is slightly distorted from the direct measurement by microscope due to the reflectance of applied pulse at the interface of 50 Ω transfer line cable and the 1 MΩ load. **Supplementary Figure 9b~d** summarized the measured amplitude and duration versus the set value. The pulse generator could certainly generate voltage pulses up to 20 V and feed them into the gate terminal of device with accurate amplitude and pulse width. And the detailed waveforms we obtained are shown in the **Supplementary Figure 10**.

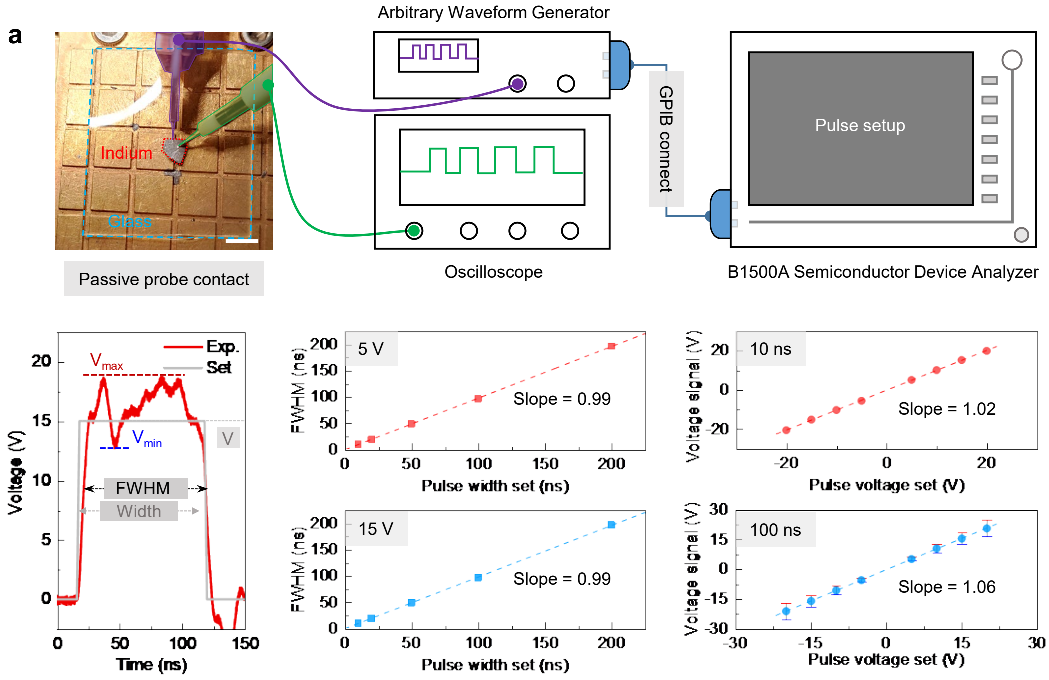

Supplementary Figure 9. Experimental setup that probes the waveform in probe station and summary of measured pulse amplitude and width. a, Configuration to probe the waveform from the adopted waveform generator. **b,** Measured pulse waveform (red) compared to the designed rectangular waveform (+15 V, 100 ns, grey). It is noted that the full width at half maximum (FWHM) of the acquired waveform is consistent with the set value, while slight peak fluctuation is observed because of the parasitic impedance. **c,** Summary about the measured FWHM of applied pulse when compared to the set value at varying amplitudes of 5 V (upper panel) and 15 V (lower panel). **d,**

Summary of the measured and set pulse amplitude at the pulse width of 10 ns (upper panel) and 100 ns (lower panel). The dash line in **c** and **d** represents the linear fitting to the measured results and set value. A slope ~1 indicate the quality of applied waveform.

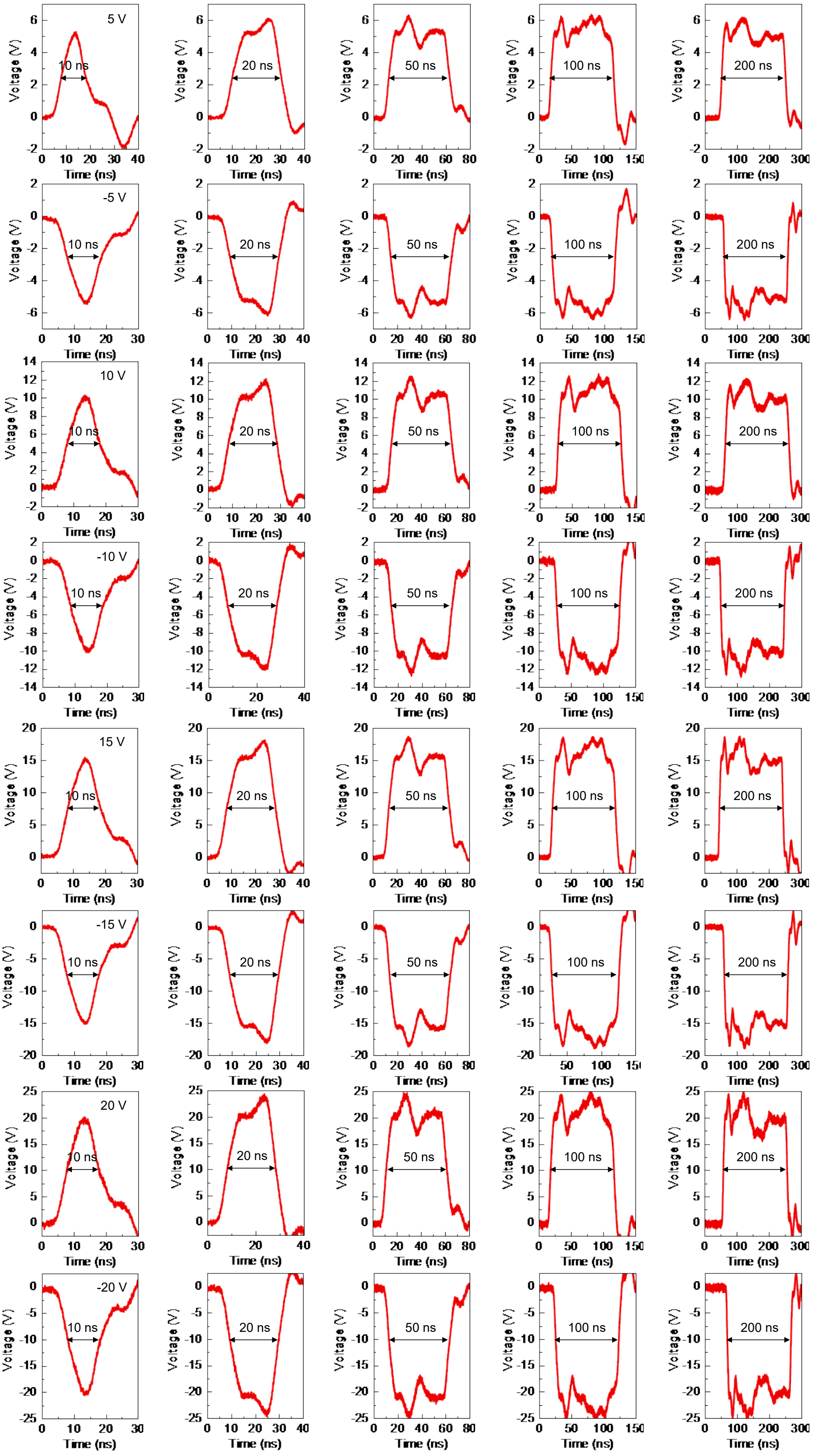

Supplementary Figure 10. Obtained pulse waveforms under different pulse amplitudes and widths. From **a** to **h**, the pulse amplitude is changed from +5 V, -5 V, +10 V, -10 V, +15 V, -15 V, +20 V and -20 V, respectively. Several pulse widths including 10 ns, 20 ns, 50 ns, 100 ns, 200 ns are for each case.

Supplementary Note 6. Tunneling carrier and barrier in memory operation

The energy band alignment across the MoS_2/hBN/FLG vdW heterostructure is illustrated in **Supplementary Figure 11.** To determine the type of carrier tunneling during memory operation, we measured the tunneling current from MoS_2 channel to graphene float gate under control gate modulation (**Supplementary Figure 12)**. If the tunneling initiates from graphene side, due to the tunable work function of graphene under gate modulation, a gate dependence of tunneling current will be observed. In experiment, the tunneling current under V_{CG}=0 V and V_{CG}=±30 V were measured in the same structure, and showed no dependence on the V_{CG} bias, which means the tunneling rate is not tuned by control gate. The phenomenon thus indicates that charge tunneling across the junction occurs by carriers from MoS_2 side, which is not tuned by bottom gate. An illustration of the carrier tunneling for memory operation is then provided in **Supplementary Figure 12c**, the hole tunneling from MoS_2 to Gr is responsible for the program operation and the electron tunneling from MoS_2 to Gr is responsible for the erase operation of the memory cell.

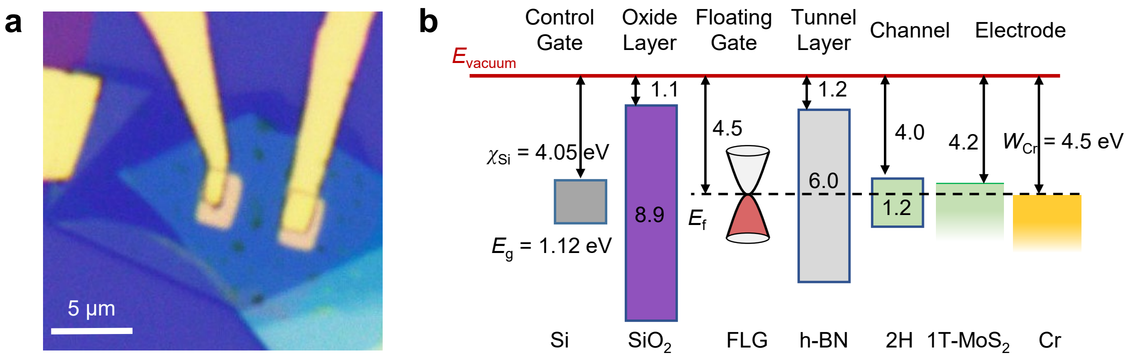

Supplementary Figure 11. Flat band alignment of the vdW heterostructure with 1T-Li_xMoS_2 edge contact. a, Optical image of an edge contacted flash memory cell. **b,** The diagram of energy band alignment in the flash memory cell in flat band condition. For a certain material, X represents the electron affinity, E_g is the band gap, E_f is the Fermi level and W is the work function.

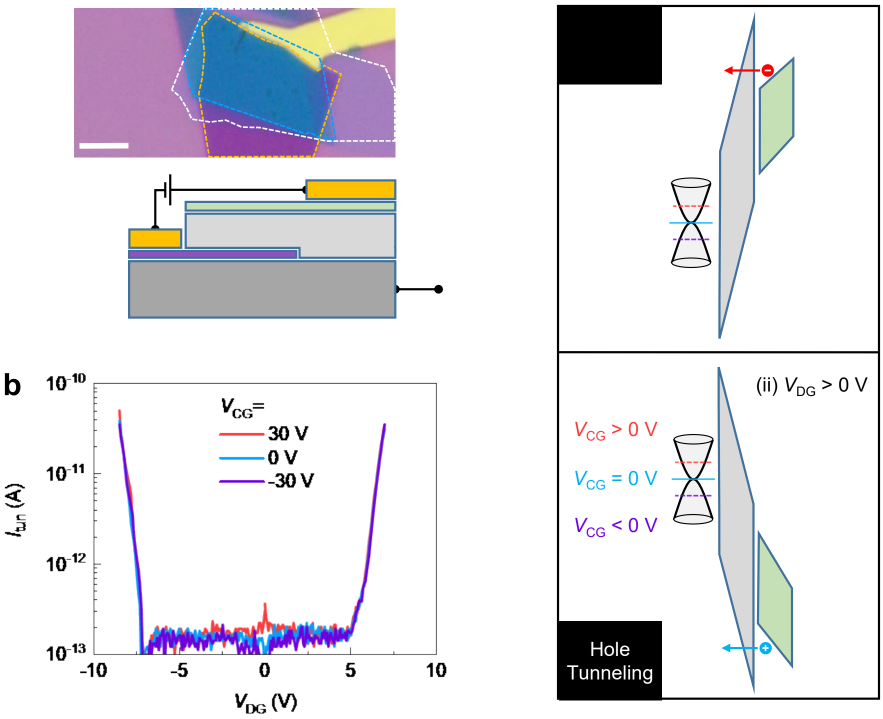

Supplementary Figure 12. Gate modulated tunneling current in MoS_2/hBN/graphene vdW heterostructure. (**a**) Optical image and the corresponding structure diagram for gate modulated tunneling current test. Tunneling current shows no dependence on the bias direction to control gate (**b**), and the corresponding band alignment (**c**) indicates that charge tunneling is from MoS_2 side.

Supplementary Note 7. Speed estimation of an ideal flash memory

Based on the Wentzel–Kramers–Brillouin (WKB) approximation, we calculated the theoretical relationship between operation voltage and speed at different conditions with reference to the method of Ref.[2]

The relationship between the operation time and pulse voltage with changing tunneling barrier from 1.0 eV to 3.0 eV are plotted in **Supplementary Figure 13a** and **b**, from which we know that the tunneling barrier height dramatically influences the operation speed for flash memory. Larger electric stress also makes it easier to achieve fast operation speed. Based on the calculation and the operation performance of our ultrafast memory cell, the relation between carrier density and pulse width under different operation voltage is simulated in **Supplementary Figure 13b**, **d.** After extract the tunneling barrier of hole (φ_h= 2.0 eV) and electron (φ_e= 2.8 eV) from **Supplementary Figure 11**, our result show the completely programing (erasing) could be achieved by a 10 ns (100 ns) pulse with the pulse amplitude of 15 V, which is in line with the experimental.

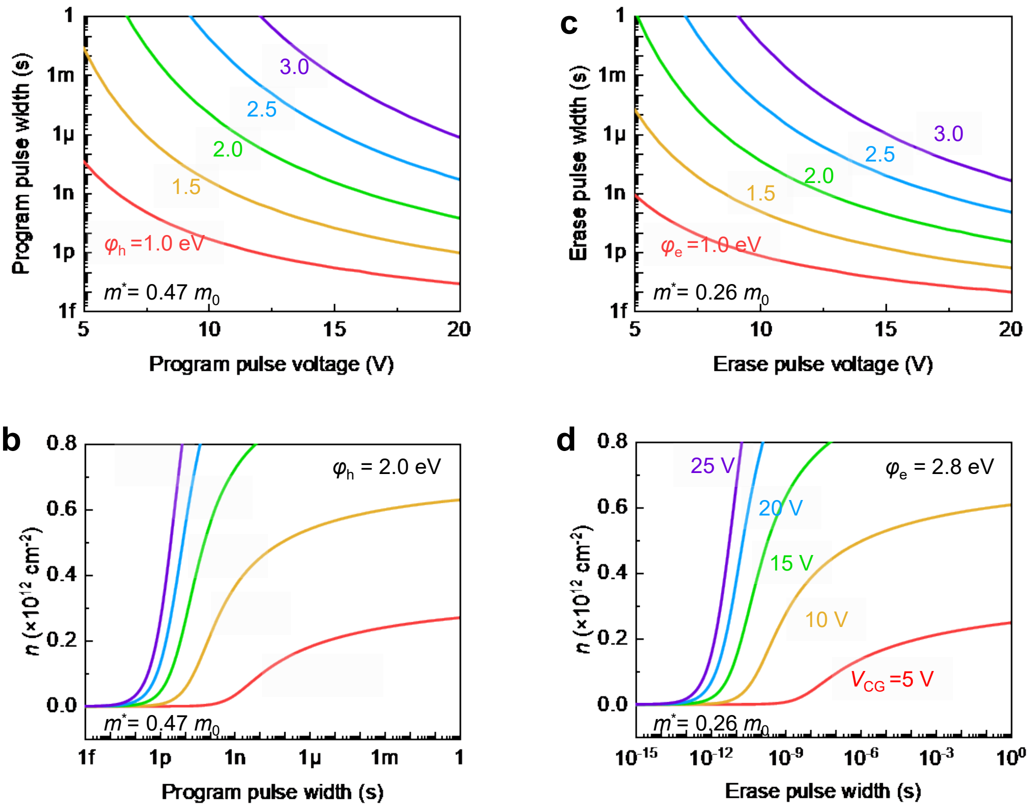

Supplementary Figure 13. Theoretical calculations of carrier ultrafast tunneling based on FN model. Theoretical relationship between the operation pulse width and voltage are calculated under different tunneling barrier height (**a** for programming and **c** for erasing). *m** is the carrier effective mass, which is 0.47m_0 for hole and 0.26m_0 for electron. **b, d,** The calculated carrier density in the floating gate layer during operation under different pulse voltage amplitude (**b** for programming and **d** for erasing), the tunneling barrier of hole/electron is 2.0/2.8 eV.

Supplementary Note 8. Estimation of gate coupling ratio

The gate coupling ratio describes the capacitance couple between control gate and float gate, and is important to efficient charge tunneling in device. To obtain the reliable GCR of present flash memory cells, we adopted two different approaches based on capacitance estimation or measured subthreshold swing (*SS*) in transistor via FG or CG, both of which reach similar GCR value~0.9 in our experiment.

Capacitance approach:

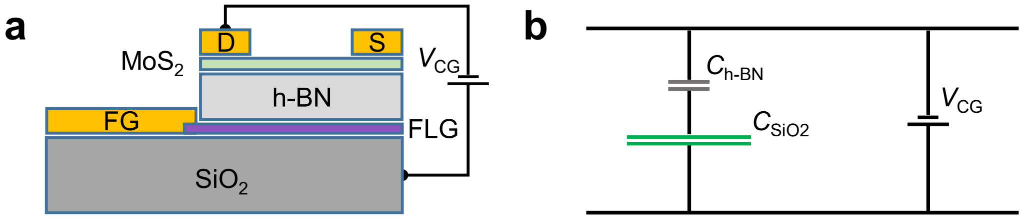

Supplementary Figure 14. Estimation of GCR from capacitive coupling area. a, Structure of the memory. **b,** Illustration of capacitance coupling in the memory, in which C_{hBN} and C_{SiO2} are the capacitance of MoS_2/hBN/FLG and FG/SiO_2/p^{++}-Si capacitors, respectively.

Using the area of floating gate and transistor channel, GCR can be directly estimated if the thickness of dielectric layer is known. **Supplementary Figure 14** illustrates the capacitance coupling in present device configuration. Based on the total capacitance C_{total} from serial connection of SiO_2 and hBN dielectric layer:

$$\frac{1}{C_{\mathrm{total}}} = \frac{1}{C_{\mathrm{hBN}}} + \frac{1}{C_{\mathrm{SiO2}}} \quad (1)$$

The GCR is written as:

$$\alpha = \frac{V_{\mathrm{FG}}}{V_{\mathrm{CG}}} = \frac{Q/C_{\mathrm{hBN}}}{Q/C_{\mathrm{total}}} = \frac{C_{\mathrm{total}}}{C_{\mathrm{hBN}}} = \frac{C_{\mathrm{SiO2}}}{C_{\mathrm{hBN}}+C_{\mathrm{SiO2}}} \quad (2)$$

Where C_{hBN} and C_{SiO2} are capacitance of MoS_2/hBN/FLG, FG/SiO_2/p^{++}-Si capacitors, respectively, *Q* is the charge stored in the series capacitor circuit. Using $C = \varepsilon\varepsilon_0 A/t$, equation (2) is further transformed into:

$$\alpha = \left(1 + \frac{\varepsilon_{\mathrm{hBN}} \cdot A_{\mathrm{hBN}} \cdot t_{\mathrm{SiO_2}}}{\varepsilon_{\mathrm{SiO_2}} \cdot A_{\mathrm{SiO_2}} \cdot t_{\mathrm{hBN}}}\right)^{-1} \quad (3)$$

where $\boldsymbol{\varepsilon_{hBN}}$ and $\boldsymbol{\varepsilon_{SiO2}}$ are the permittivity of hBN and SiO_2, respectively, A_{hBN} and A_{SiO2} are the overlap area of MoS_2/hBN/FLG, FG/SiO_2/p^{++}-Si capacitors, respectively, t_{hBN} and t_{SiO2} are the thickness of hBN flake and SiO_2 layer, respectively. Notably, the float gate is extended by metal contact and has larger area than the transistor channel and FLG, which makes $A_{hBN} \ll A_{SiO2}$ (see **Supplementary Table 1**). Thus, our flash memory cells tend to have GCR factors near unity.

Subthreshold swing approach:

The *SS* value in transistor reflects the coupling between transistor channel and gate modulation, and is influenced by the gate capacitance and the present of interface defects. With a commen transistor channel, GCR value can be estimated by comparing the observed *SS* under FG and CG regulation.

Experimentally, the values of the (*SS*) were calculated in the thermionic regimes of the transfer curves according to:

$$SS = \left[\frac{\mathrm{dlog}(I_{\mathrm{ds}})}{\mathrm{d}V_{\mathrm{G}}}\right]^{-1} \quad (4)$$

where I_{ds} is the channel current measured in the thermionic regime, V_{G} is the applied gate bias.

When there is no Fowler-Nordheim tunneling taking place, the voltage applied on the control gate is portionly coupled to the floating gate, so the gate coupling ratio can be estimated from the subthreshold swing in floating-gate transfer curves and control gate transfer curves from Equation (5):

$$SS_{\mathrm{FG}} = \left[\frac{\mathrm{dlog}(I_{ds})}{\mathrm{d}V_{FG}}\right]^{-1} = \left[\frac{\mathrm{dlog}(I_{\mathrm{ds}})}{\mathrm{d}(\alpha\cdot V_{\mathrm{CG}})}\right]^{-1} = \alpha \cdot SS_{CG} \quad (5)$$

where SS_{FG} and SS_{CG} are subthreshold swings in floating-gate transfer curves and control gate transfer curves, respectively, V_{FG} and V_{CG} are the applied floating gate voltage and control voltage, respectively, I_{ds} is the channel current measured when V_{ds} is fixed at 0.1 V. **Supplementary Figure 15** shows the transfer curves of different flash memory devices under regulation of floating gate and control gate. The maximum FG sweeping voltage of each device are chosen at small value to avoid tunneling occurring, and the SS_{FG} and SS_{CG} are extracted at a consistent current range of each device.

The calculated gate coupling ratios were presented in the **Supplementary Table 1**. The estimation based on capacitance or subthreshold swing approach yield similar values ~0.9.

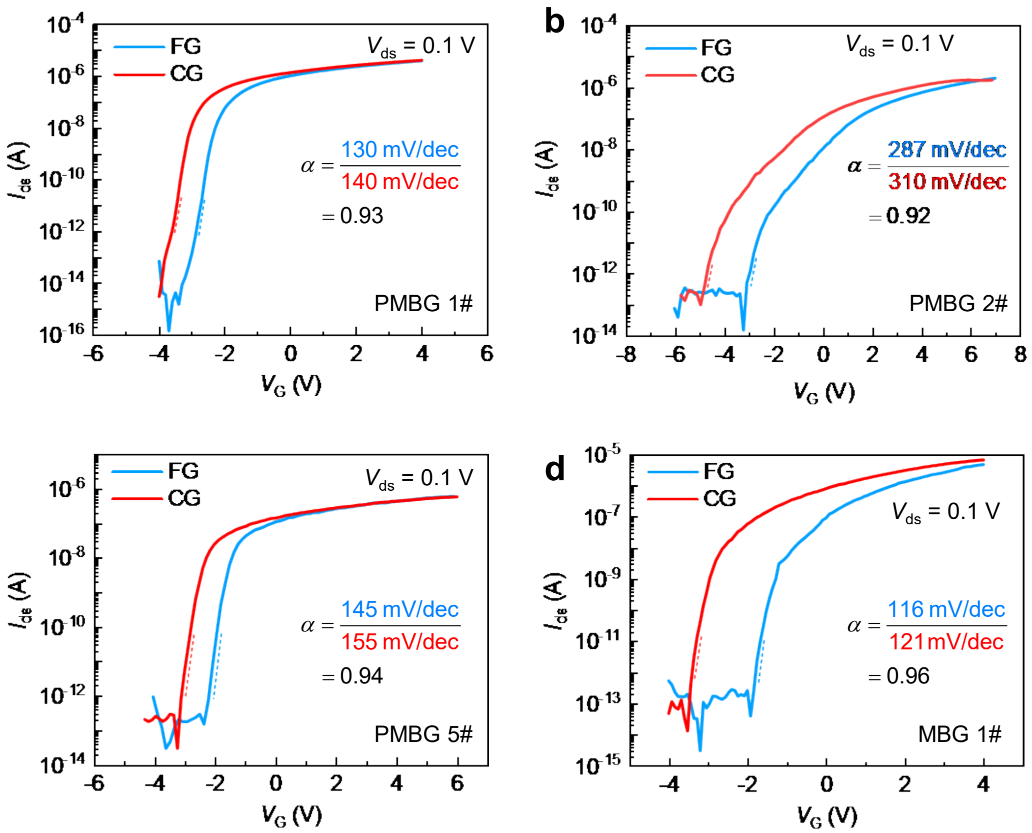

Supplementary Figure 15. Estimation of GCR from subthreshold swing approach for four different devices. a, b, c, d: PMBG1#, PMBG2#, PMBG5# and MBG1#, respectively**.** Identical gate modulation and peak *SS* value is obtained from the transfer curves measured when varying V_{FG} or V_{CG}, indicating high GCR for all devices.

Supplementary Table 1. Comparison of the determined GCR in several devices using area or subthreshold swing approach.

Device	SS_{FG} (mV/dec)	SS_{CG} (mV/dec)	α_{ss}	A_{hBN} (μm^2)	t_{hBN} (nm)	A_{SiO2} (μm^2)	t_{SiO2} (nm)	α_A
PMBG 1#	130	140	0.93	136	14.5	19250	300	0.89
PMBG 2#	287	310	0.92	70.5	11.3	15623	300	0.91
PMBG 5#	145	155	0.94	167	12.8	16080	300	0.83
MBG 1#	116	121	0.96	125	10.1	19256	300	0.86

Supplementary Note 9. Validation of the role of edge contact using paired memory cell

In experiment, paired memory cells are made to compare the role of edge and top contact using the same vdW heterostructure. This avoids the uncertainty of quality and thickness variation among samples. The thickness evaluation of paired memory cell in Figure 2 is provided in **Supplementary Figure 16.**

In addition to results discussed in Figure 2, we also fabricated paired memory cells that has both edge contact, but with or without overlap between contact and float gate. The results displayed in **Supplementary Figure 17** further indicate that keeping the edge contact under float gate modulation is critical for a fast P/E speed. When an additional access region is present in device between the contact and the float gate modulation, the Schottky barrier at edge contact is not tuned during memory operation, and the advantage in P/E speed disappears.

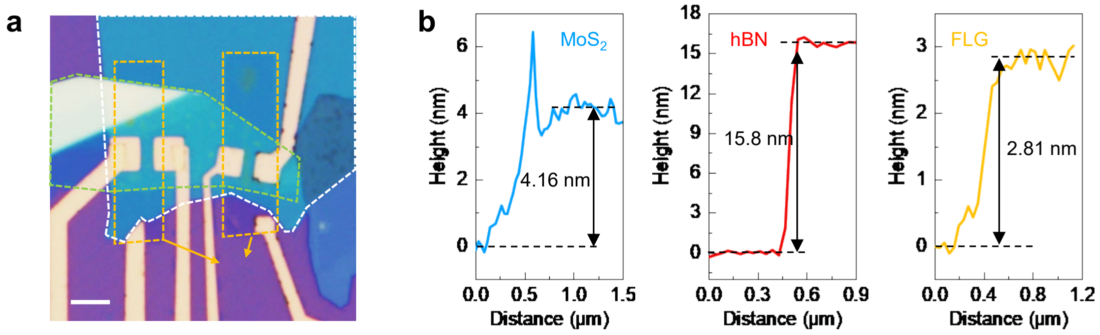

Supplementary Figure 16. Thickness characteristics of the paired FLG/hBN/MoS_2 heterostructure. a, Optical microscope image of the paired FLG/hBN/MoS_2 memory cells. The boundaries of FLG, hBN and MoS_2 are marked by yellow, white and green dashed lines, respectively. The scale bar is 5 μm. **b**, Thickness of MoS_2 (blue), hBN (red) and FLG (yellow)respectively.

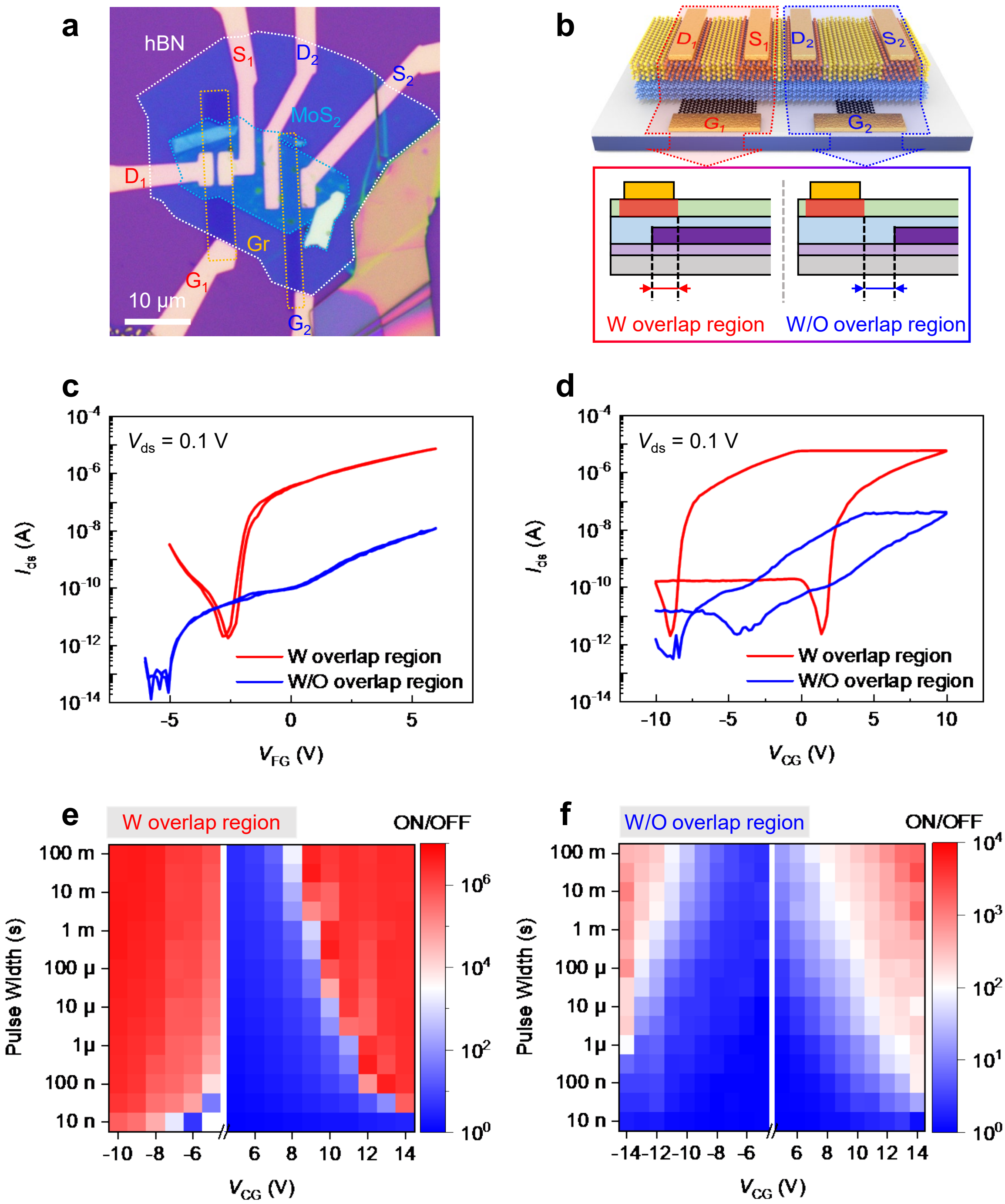

Supplementary Figure 17. Comparison of the P/E performance of memory cells with and without overlap between float gate and 1T contact. (**a, b**), Optical microscope image (**a**) and schematic illustration (**b**) of the paired memory cells on the same vdW heterostructure. (**c, d**), Transfer curves under floating gate (**c**) and control gate (**d**) modulation. (**e, f**), Map of the attained ON/OFF ratio of memory under different voltage pulse conditions when changing both the amplitude and pulse width: 1T edge contact with overlap region (**e**) and without overlap region (**f**)

Supplementary Note 10. P/E heat map for additional edge contacted memory cells.

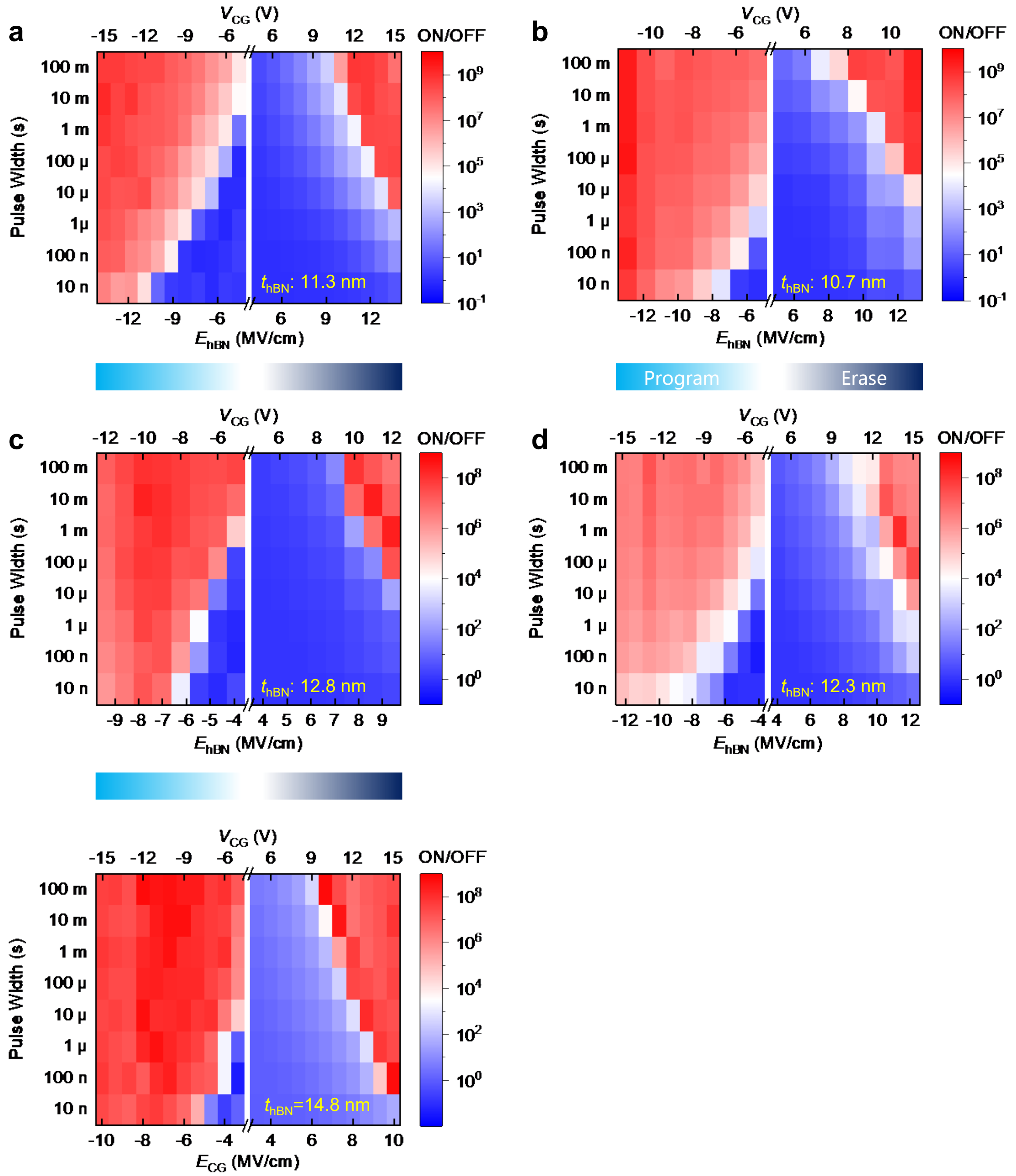

Supplementary Figure 18. The P/E heatmaps of other edge contacted flash memory devices under variable pulse amplitudes and durations. a, b, c, d, e, The heatmaps of ON/OFF ratio distribution with respect to pulse width and field strength. The operation voltage V_{CG} on the upper axis corresponds to the electric field E_{hBN} on the lower axis, and t_{hBN} is the thickness of hBN in the devices.

Supplementary Note 11. Evaluation of tunneling current density in differently configured heterostructure

If compared to tunneling from 2H-MoS_2's valance band, the tunneling from 1T-MoS2 contact through hBN has higher barrier (**Supplementary Figure 19**). Same case exists for tunneling from Cr/Au contact though hBN. Thus, we expect the overlap region of metal contact (1T or Cr/Au) with bottom graphene float gate contribute little to the tunneling current, and the memory operation. However, the presence of lateral charge injection pathway from 1T edge contact to 2H-MoS2 does enhance the measured tunneling current density if compared to conventional top contact, under the vertical electric field (8 MV/cm) in hBN (**Supplementary Figure 20**). This validates the role of edge contact in enhancing operation speed of memory cell.

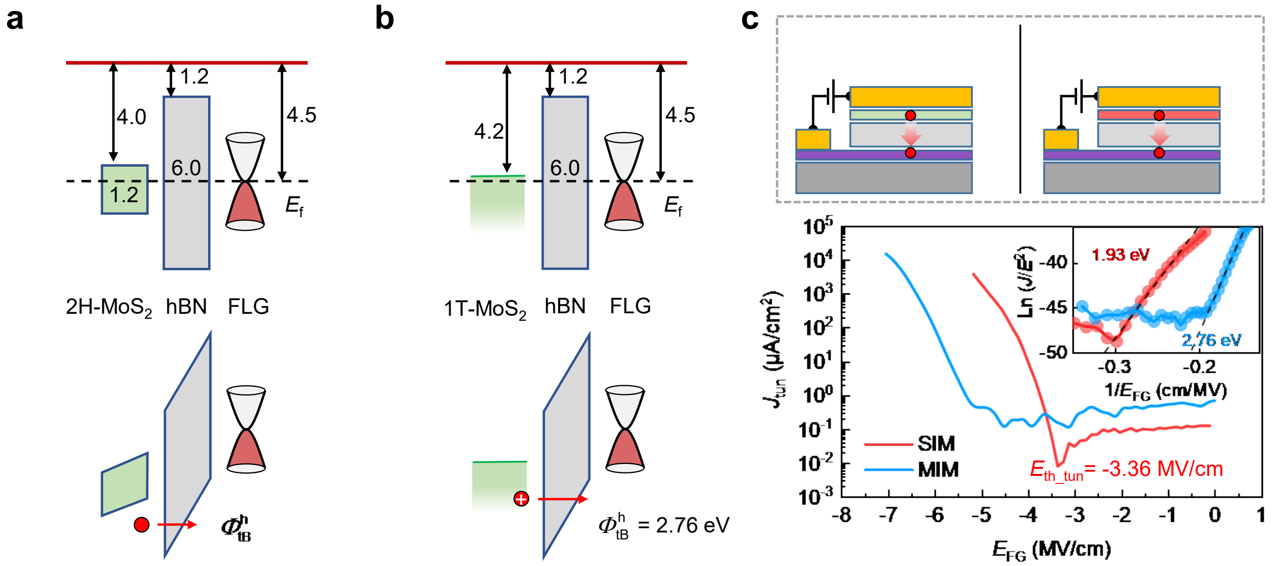

Supplementary Figure 19. Band alignment and tunneling current of 1T-Li_xMoS_2/hBN/Graphene, and 2H MoS_2/hBN/graphene. Holes tunneling from 1T-Li_xMoS_2 through hBN has higher energy barrier (**a**,**b**) and lower current (**c**) than from the conduction band (CB) or valance band (VB) of 2H-MoS_2 at the negative bias to graphene layer due to the presence of band offset between 1T-Li_xMoS_2 and 2H-MoS_2.

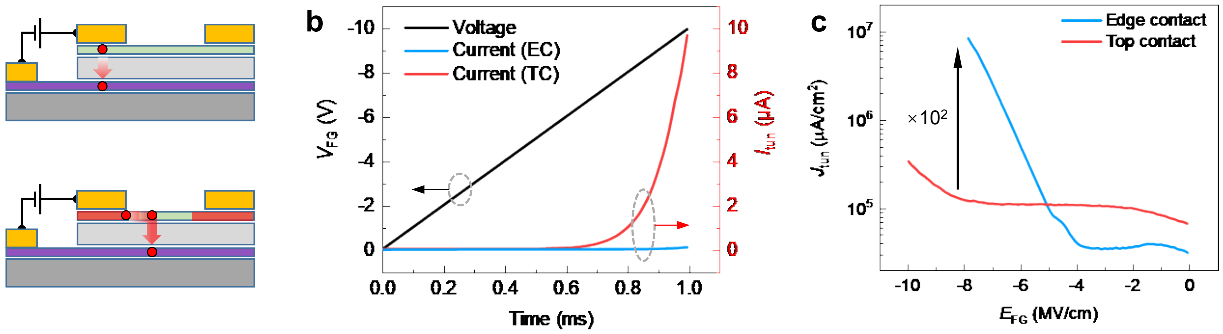

Supplementary Figure 20. Comparison of the transient tunneling current of memory cells with edge and top contact. a, The schematic diagram of the transient tunneling current test. **b**, The demonstration of the transient voltage applied and current monitored. The vertical voltage is applied within 1 ms and the monitor of the corresponding tunneling is also finished, and the comparison of tunneling current density are normalized according to the device area (**c**).

Supplementary Note 12. Effect of float gate choice to memory operation

A symmetric heterostructure of edge-MoS_2/hBN/MoS_2-top was constructed to support the speed enhancement effect of edge contact. It is apparent in the following **Supplementary Figure 20** that the device exhibit highly asymmetric P/E speed. The memory cell can be programmed within 100 ns, but can be only erased with >100 ms pulses. Such highly asymmetric P/E behavior in the symmetric heterostructure with only contact difference indicated the critical role of contact mode. The result can be properly understood using hot hole injection via the highly tunable edge contact, since the injection via top contacted 2H-MoS_2 is significantly impeded by the access region from metal contact

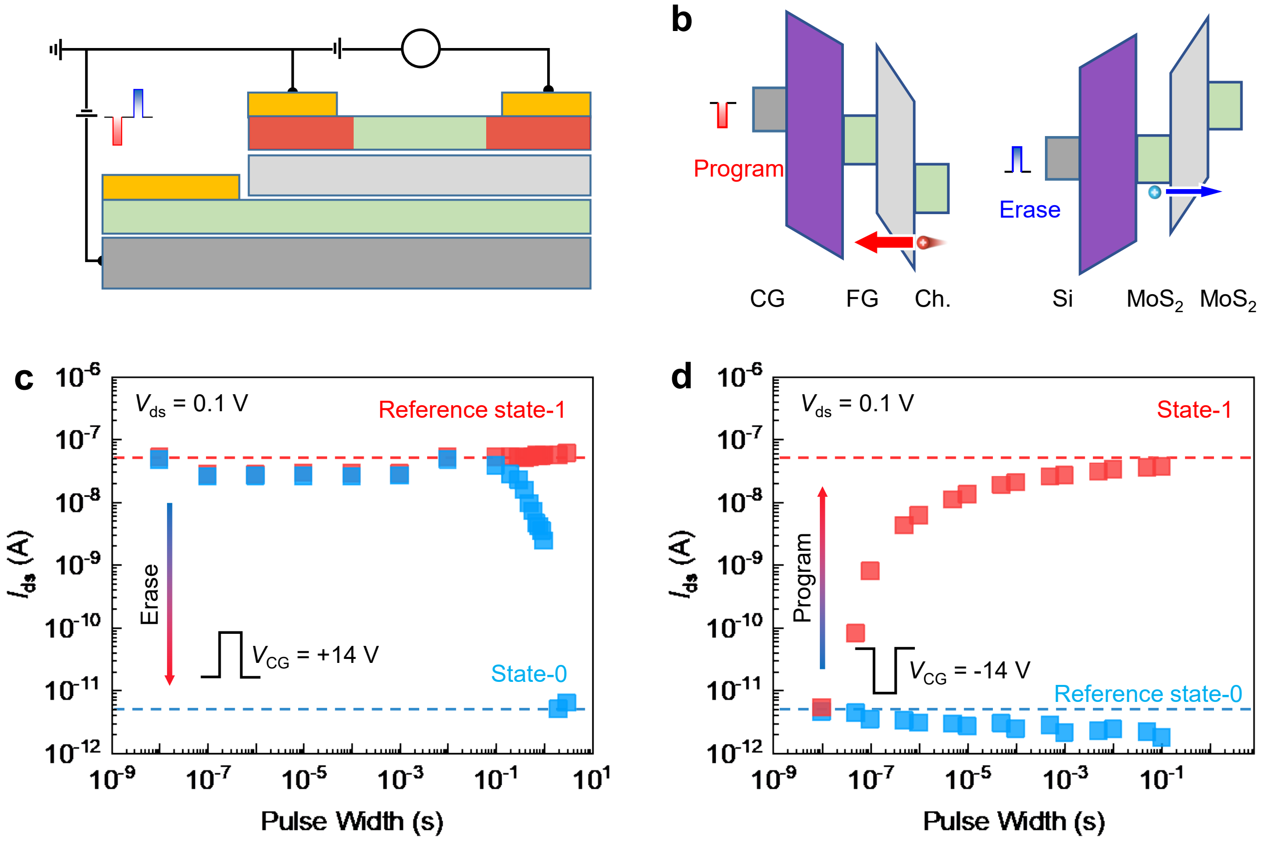

Supplementary Figure 21. Edge contacted flash memory with MoS_2 as the float gate. a, Configuration of the memory. **b**, Energy band diagram of the memory under program (left)/erase (right) operation. **c** and **d** reveal the program and erase performance when varying the width for applied V_{CG} pulse (-14 V for program, and 14 V for erase). The reference states (state-1 in **a** and state-0 in **b**) were set by initializing voltage pulses with 100 ms width. Compared with the program operation speed (100 ns), the erase operation speed (>100 ms) is slower.

Supplementary Note 13. Estimation of trap density in memory cells

To further characterize the defect concentration in devices with edge and conventional top contact, we use the high-low frequency method based on the measured capacitance-voltage (C-V) characteristic in the frequency range of 4 kHz to 100 kHz.[3, 4] The experimentally measured capacitance per unit area C'_{p} is consisted of:

$$C'_{\mathrm{p}} = \left(\frac{1}{C'_{\mathrm{S\_MoS2}} + C'_{\mathrm{it}}} + \frac{1}{C'_{\mathrm{g}}}\right)^{-1} \quad (6)$$

Where $C'_{\mathrm{S\_MoS2}}$ is the semiconductor capacitance of MoS_2, C'_{it} is the capacitance of traps and C'_{g} is the geometric capacitance related to hBN layer and the vdW gap, all the capacitance are normalized by area. Since the interface traps tend to response slowly compared to $C'_{\mathrm{S\_MoS2}}$ and C'_{g}, the interface trap density D_{it} can be estimated by:

$$qD_{\mathrm{it}} = \left[\left(\frac{1}{C_{LF}} - \frac{1}{C_{\mathrm{hBN}}}\right)^{-1} - \left(\frac{1}{C_{\mathrm{HF}}} - \frac{1}{C_{\mathrm{hBN}}}\right)^{-1}\right] \quad (7)$$

Where C_{LF} and C_{HF} are respectively the areal capacitance at low (4 kHz) and high (100 kHz) frequency limit, and q the elementary charge. In our structure, the geometric capacitor C'_{g} is comprised of C'_{hBN} and C'_{in}, corresponding to the geometric capacitance of hBN and the interlayer capacitance, as described by $C'_{\mathrm{g}} = (1/C'_{\mathrm{hBN}} + 1/C'_{\mathrm{in}})^{-1}$, where C'_{in}~25.3 μF/cm^2 obtained from Ref.[4], and C'_{hBN} is calculated according to its thickness. Because C'_{in} is significantly larger than C'_{hBN}, it can be neglected in calculation without influencing the result.

Figure 3c displays the measured C-V characteristics of both devices. The apparent decrease of C'_{p} when increasing frequency reflects the effect of trap states. To properly compare the trap distribution, we firstly extracted the flat band potential according to Mott-Schottky plot in **Supplementary Figure 22a** and **c**. Based on equation (7), the energy distribution of trap density is determined and shown in **Supplementary Figure 22b** and **d**. By integrating the trap density over the measured voltage range, the trap density for edge and top contacted memory cell were determined to be 0.8x10^{12} cm^{-2} and 2.1x10^{13} cm^{-2}.

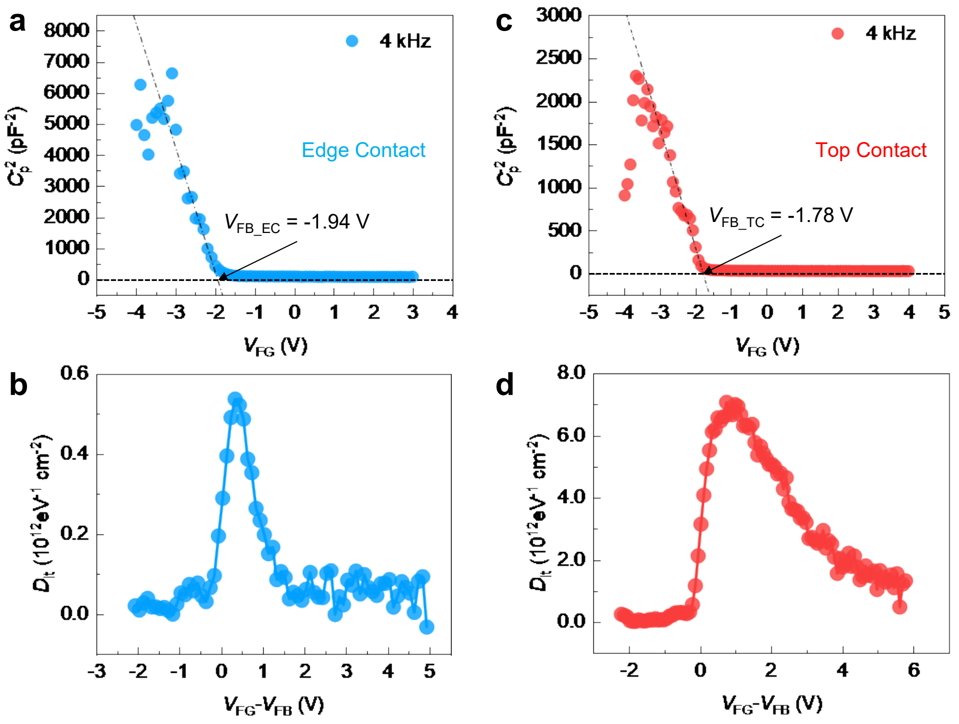

Supplementary Figure 22. Determination of trap density in edge and top contacted memory cells using C-V characteristics. Mott-Schottky plot and extracted trap density using high-low frequency method for edge (a, b) and top contacted memory cell (c, d).

Supplementary Note 14. Potential sources of trap states in device fabrication

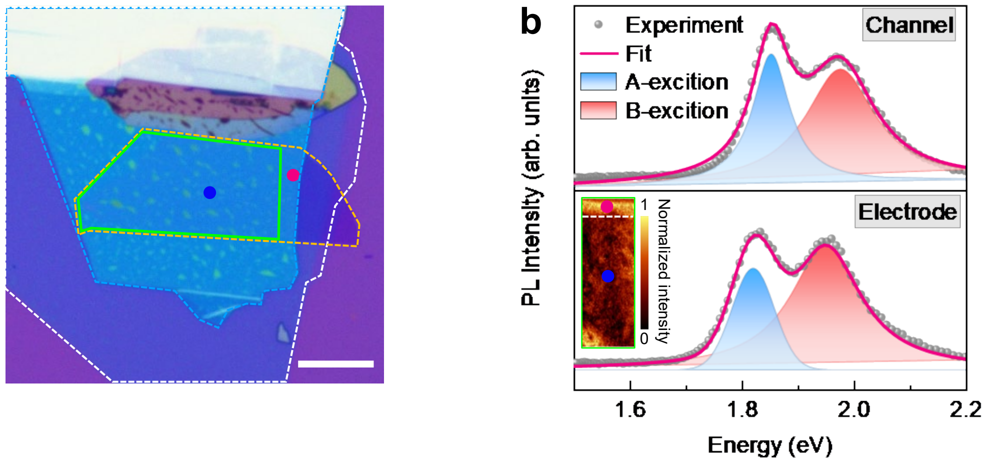

In addition to metal induced gap states (MIGS), the high trap density under contact may be related to the vulnerable lattice of 2D MoS_2 to electron beam irradiation and metal evaporation during device fabrication[5, 6]. Measurement of spatial resolved photoluminescence spectra in device supported that the irradiation by electron beam could induce traps in pattern contact area. As indicated in **Supplementary Figure 23**, if compared to the channel area without irradiation, the PL spectra at the defined contact area exhibit shift of peak position and decrease of intensity, which suggests the increase of trap density.

Supplementary Figure 23. Electron beam effect on defect generation under Cr top contact. a, Optical microscope image of the vdW MoS_2/hBN/FLG heterostructure. MoS_2 flake is marked by blue dashed lines, and the irradiation area is marked by green dashed lines. **b,** The PL spectrum at the selected pots in **a**. The inset shows the PL mapping image (655–685 nm) of the MoS_2 flake in **a**. The blue pot for the MoS_2 with electron beam irradiation, and red pot for without irradiation.

During the fabrication of metal contacts by thermal evaporation, the impinging metal atoms inevitably takes damage to the MoS_2 under the electrodes[7, 8], which is reported to be an obvious source of defects in devices based on two dimensional materials. According to the cross-section transmission electron microscope (TEM) study, as shown in **Supplementary Figure 24**, we also found notable layer broken and defects were presented in MoS_2 under the electrode region, while a comparative high-quality MoS_2 interface could be seen in the channel region.

In brief, during the fabrication of Cr contact using EBL processes, defects could be generated under the contact area from both electron beam irradiation and electrode deposition processes. In comparison, the impact of these defects is avoided by transforming the affected area into a metallic 1T phase.

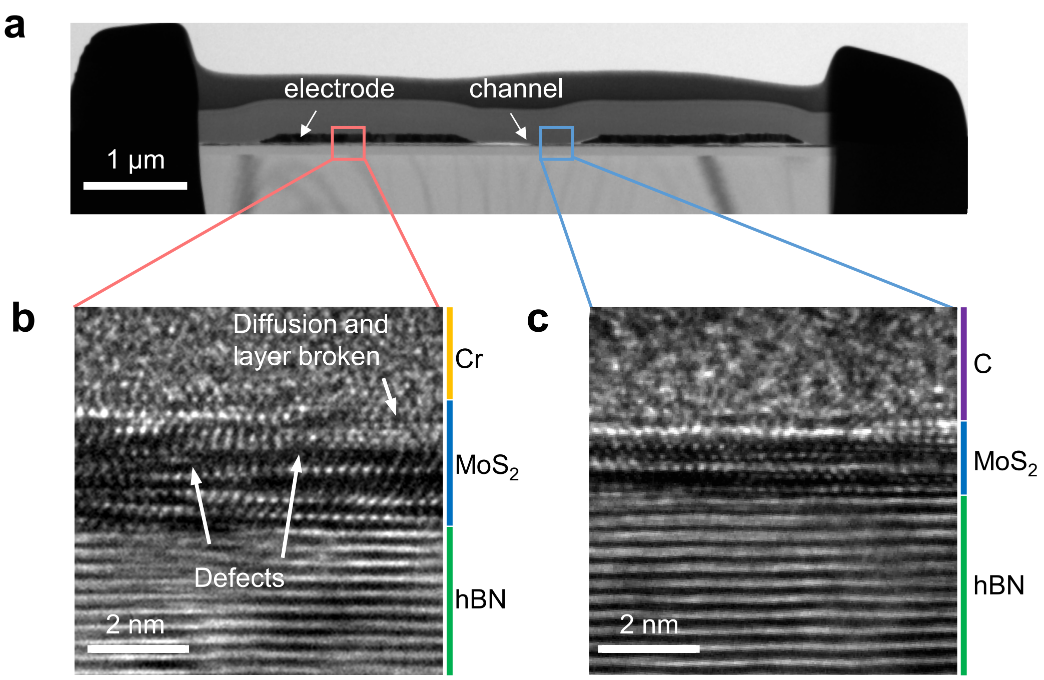

Supplementary Figure 24. TEM characterization for MoS_2/hBN/FLG vdW heterostructure. a, Cross sectional piece structure carved by focused ion beam (FIB) of the Cr top contacted flash memory device. **b, c,** The cross-section TEM images of the MoS_2/hBN/FLG heterostructure under the deposited Cr/Au electrode and in the channel area (labeled in **a**).

Supplementary Note 15. Extraction of Schottky barrier at contact

Temperature-dependent transfer curves under the regulation by V_{FG} were measured to compare the effective Schottky barrier height of the top and edge contacted devices, as shown in **Supplementary Figure 25a, b**. The effective barrier height Φ_{SB}^{e} is extracted by the following thermionic emission equation[9]:

$$I_{\mathrm{ds}} = AT^2 \exp\left(\frac{q\Phi_{SB}^{\mathrm{e}}}{kT}\right)\left[1 - \exp\left(\frac{qV\mathrm{ds}}{kT}\right)\right] \qquad (8)$$

where I_{ds} is the current through the channel, A is the Richardson's constant, T is the temperature, q is the charge of electron, V_{ds} is the voltage applied on drain electrode, k is the Boltzmann constant. **Supplementary Figure 25c, d** displays the fitting to experimental results for top contacted and edge contacted memory cell when varying V_{FG}, from what the gate tunable Schottky barrier is further extracted in **Supplementary Figure 26.**

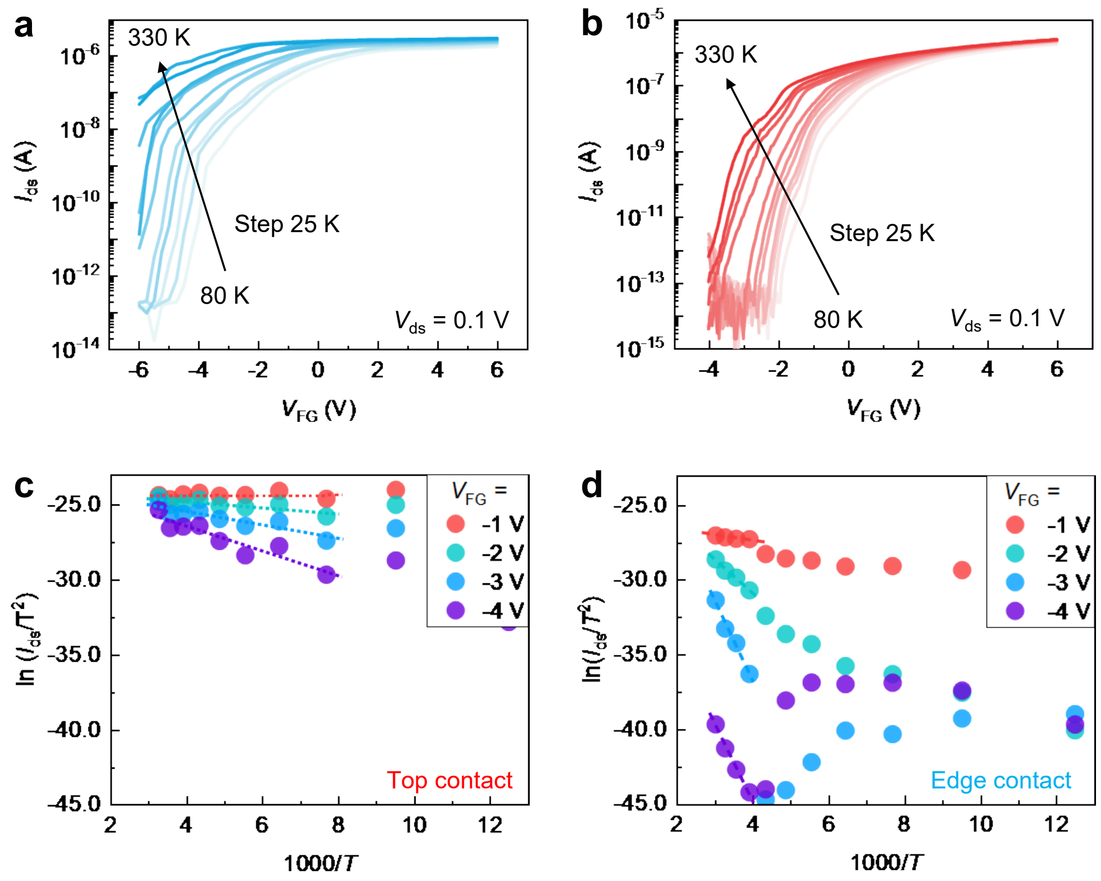

Supplementary Figure 25. Comparison of temperature-dependent transfer curves with floating gate in device MBG1# and PMBG8# and the Arrhenius fitting curves for the extraction of Schottky barrier height. a, b, The transfer curves of the top/edge contacted flash memory devices while the temperature changes from 80 K to 330 K with a step of 25 K. **c, d**, Arrhenius fitting curves at different floating gate voltages in device MBG1# and PMBG8#, respectively.

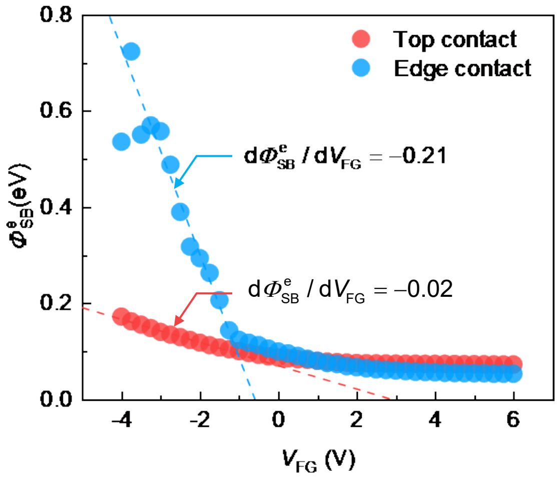

Supplementary Figure 26. The extracted effective barrier height (Φ_{SB}^{e}) as a function of applied floating gate voltages for top contact and edge devices, and the FLP factor was extracted.

Supplementary Note 16. Temperature accelerated retention measurement

To properly evaluate the retention lifetime of our memory cell, Arrhenius plot method was used. The V_{th} drift with time for on and off states after program and erase were measured to first determine the acceleration ratio at varied temperature. **Supplementary Figure 27a** displays the V_{th} drift at different temperatures. The retention lifetime is considered as when the extrapolation of V_{th} of state-0 and state-1 crosses. Based on the temperature dependent retention lifetime (**Supplementary Figure 27c**), we determined an activation energy E_a=1.95 eV for present memory cell, according to the fitting to experimental data using the Arrhenius equation[10]:

$$t_\mathrm{T} = t_\mathrm{R} \exp\left(\frac{E_\mathrm{a}}{kT}\right) \quad (9)$$

where t_T is the retention lifetime at field temperature retention, t_R is the reference retention time corresponding to the infinite temperature, E_a is the activation energy, k is Boltzmann's constant, T is the field temperature. We plotted a fitting curve and back calculated the critical field temperature of 78.6 °C for 10-year retention.in **Supplementary Figure 27c.**

According to the activation energy we extracted, the data retention time at low temperature could be calculated follows the acceleration function[11]:

$$AR = \frac{t_\mathrm{L}}{t_\mathrm{H}} = \exp\left[\frac{E_\mathrm{a}}{k}\left(\frac{1}{T_\mathrm{L}} - \frac{1}{T_\mathrm{H}}\right)\right] \quad (10)$$

where AR is the acceleration ratio, T_L and T_H are the application temperature (usually at low temperatures) and accelerated bake stress temperature (usually at high temperatures). The acceleration ratio for room temperature (RT) under different temperature is calculated in **Supplementary Figure 27d**.

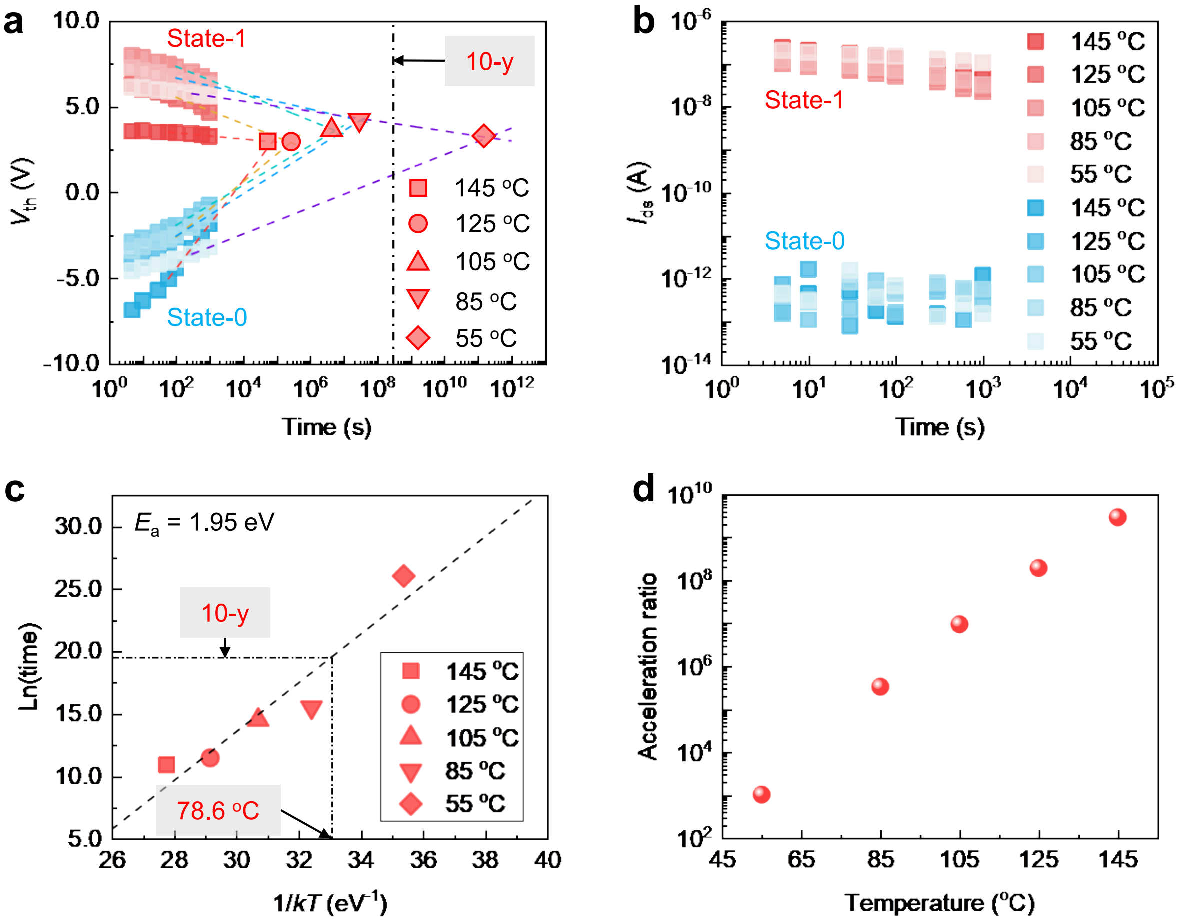

Supplementary Figure 27. Temperature-dependent retention performance and the extraction of activation energy by Arrhenius plot method. a, Retention performance at five different temperatures (55, 85, 105, 125, 145 °C) for edge contacted flash memory device PMBG17#. **b,** Retention-failure evaluation of the device from the V_{th} at different temperatures. **c,** Arrhenius fitting and extrapolation for the retention lifetime. **d,** The calculated acceleration ratio under different temperature.

Supplementary Note 17. Retention behavior during cycled endurance measurement

Flash memory cells are prone to degrade under stress effect, which is known to induce defects and charge trapping in dielectric layer, thereby changing the tunneling efficiency and also increase the leakage current that influences both the operation speed and retention. In our case, the memory cell exhibits asymmetric P/E efficiency, which are likely to cause over-program if identical amplitude is chosen for both program and erase. As indicated in **Supplementary Figure 28a(i)-(iii)**, after repeated cycling with P/E pulses of identical 15 V amplitude, the resulted state-0 tend to drift to higher conductance. It is worth noting that the stress effect can be relived after a while, and we attribute the state drift to positive charge trapping in hBN layer that can be gradually released when the memory cell is in idle state.

To avoid significant stress from program operation, we chose to reduce the amplitude of applied P/E pulse while keeping a ON/OFF ratio >10^5 and ultrafast speed (10/100 ns for P/E). As indicated in **Supplementary Figure 29a(iv)**, stable ON/OFF state is obtained before abrupt failure of the memory. From the experiment, we found that after 233 k cycles, the memory still manifested excellent retention characteristics even under temperature acceleration, as indicated in **Supplementary Figure 29b**.

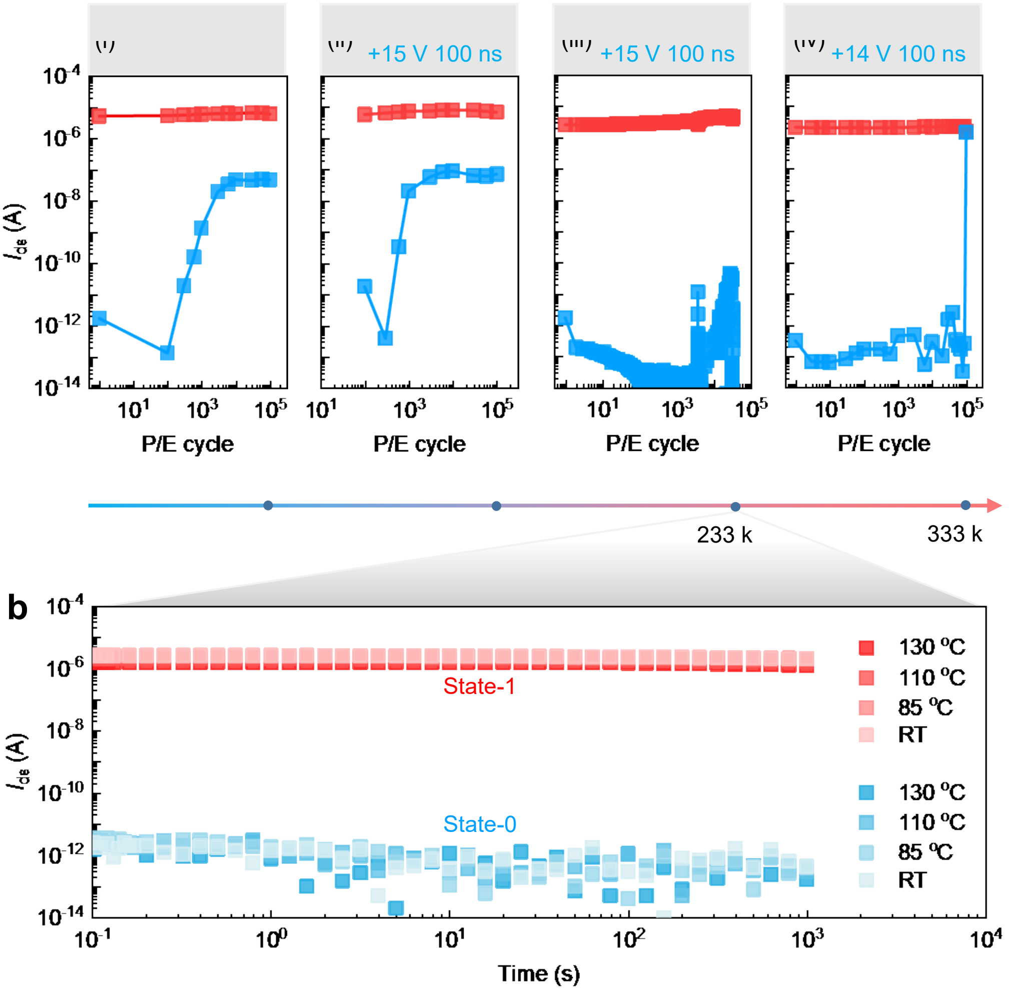

Supplementary Figure 28. Endurance performance of memory cell PMBG1#. a, The endurance performance under different P/E pulse conditions. The device failed after more than 330k cycles. **b,**

The retention characteristic of PMBG1# after 233 k P/E cycles. The readout current at state-0 and state-1 displays excellent stability at RT, 85, 110 and 130 ^{o}C.

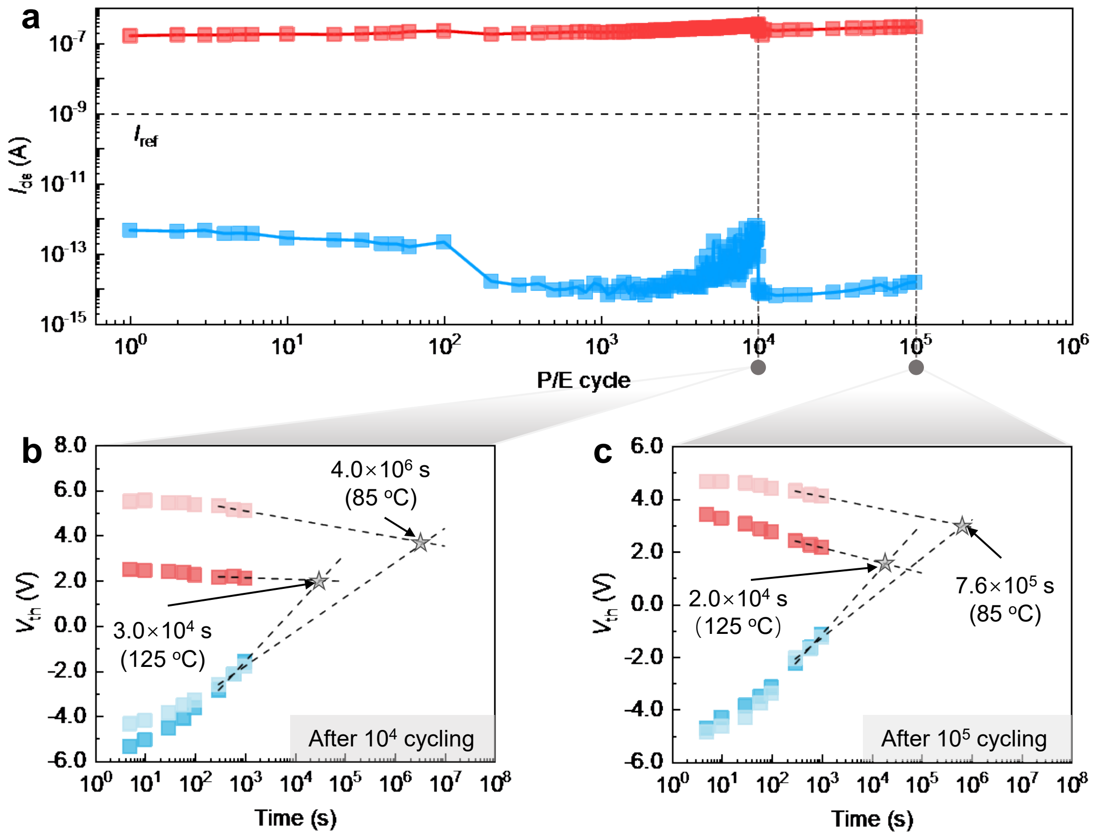

Supplementary Figure 29. Post-cycling data retention at different temperature. a, The endurance cycling test of the edge contacted flash memory device PMBG17#. **b**, **c**, Data retention test base on V_{th} at 85 °C and 125 °C after 10^4 and 10^5 P/E cycles, respectively. The estimation of retention lifetime using extrapolation method is marked for each temperature conditions.

Supplementary Note 18. Comparison of endurance lifetime with edge and top contact configuration

The superior endurance lifetime of edge contacted memory cell is confirmed by comparing to top contacted memory cell fabricated in paired structure. As shown in **Supplementary Figure 30**, top contacted memory cell has typical lifetime ~10^4 cycles, while the one with edge contact could tolerate 10^6 P/E cycles.

In view of the different endurance lifetime of top and edge contacted memory cells, their optical images before and after device failure is compared for top contacted (MBG12#) and edge contacted (PMBG1#). As shown in **Supplementary Figure 31**, apparent flake damage was observed around the contact region in top contacted memory cell MBG12# (**Supplementary Figure 31b**). This is understood as a result of the high defect density under contact affected area (CA), which under strong electric field would migrate and lead to locally focused electric field that deteriorate tunneling dielectric layer.[12] In comparison, we observed no visible damage in memory cell PMBG1# (**Supplementary Figure 31e**), because of well suppressed defects at the contact area. According to separate time-to-breakdown analysis (**Supplementary Figure 32**), the heterostructure in edge contact configuration is also more robust than the one in conventional top contact configuration.

In experiment, the superior endurance lifetime of edge contacted memory cell was confirmed in multiple devices (**Supplementary Figure 33**).

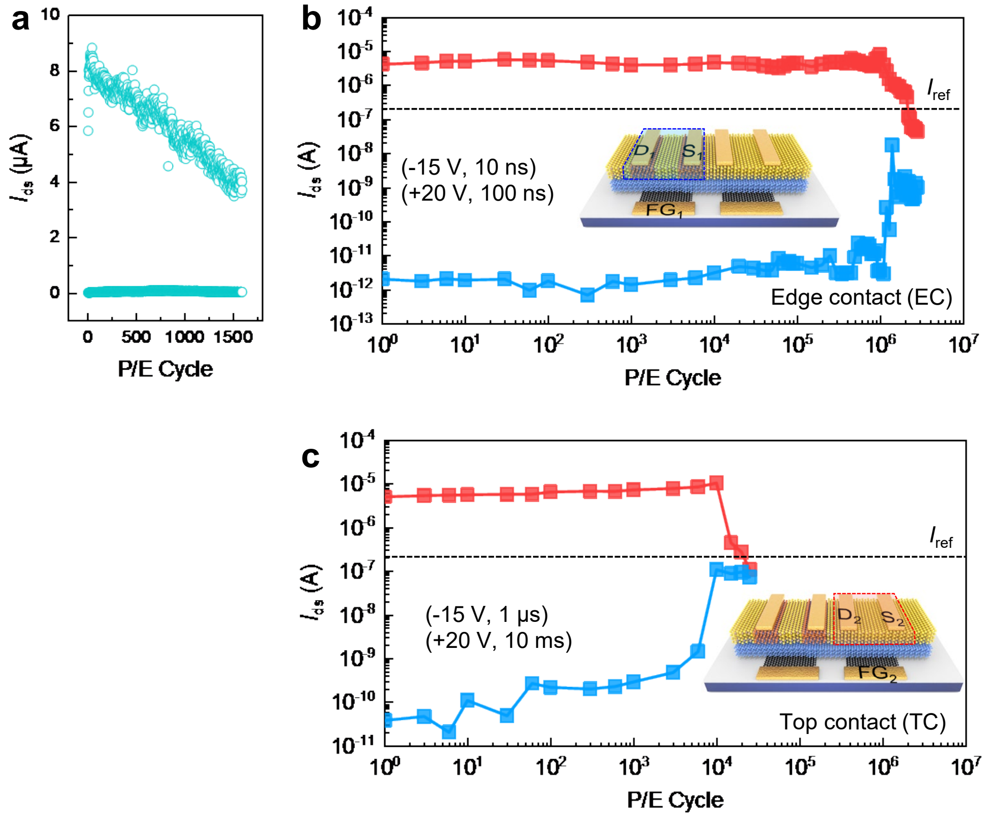

Supplementary Figure 30. Endurance performances of paired memory cells. Compared to edge contacted memory cell **(a, b)**, top contacted memory cell **(c)** working at same operation voltage requires longer pulse duration to reach the same on/off ratio, and results in low endurance lifetime ~10^4 to 10^5 cycles. For edge contacted memory cell, the memory states at first 1.5k cycles were read after each P/E operation, it is then switched to periodic reading for endurance lifetime >10^4 cycles.

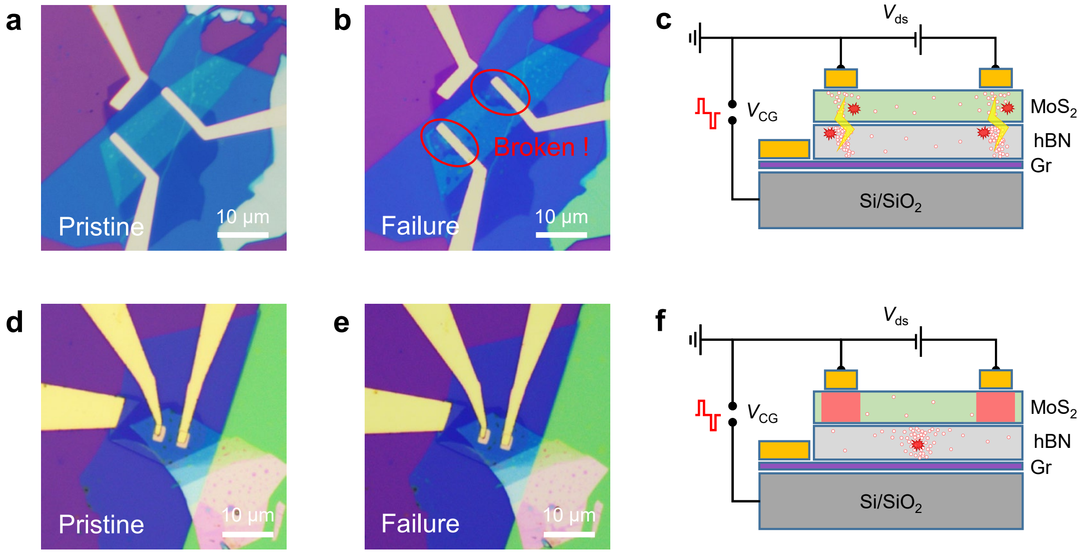

Supplementary Figure 31. Failure analysis of devices. Comparison of the optical images before and after device failure for top contacted (**a**, **b** for MBG12#) and edge contacted (**d**, **e** for PMBG1#) memory cells. **c, f** are the schematic diagrams illustrating the failure scheme for top contacted and edge contacted devices by dielectric breakdown of hBN layer, respectively.

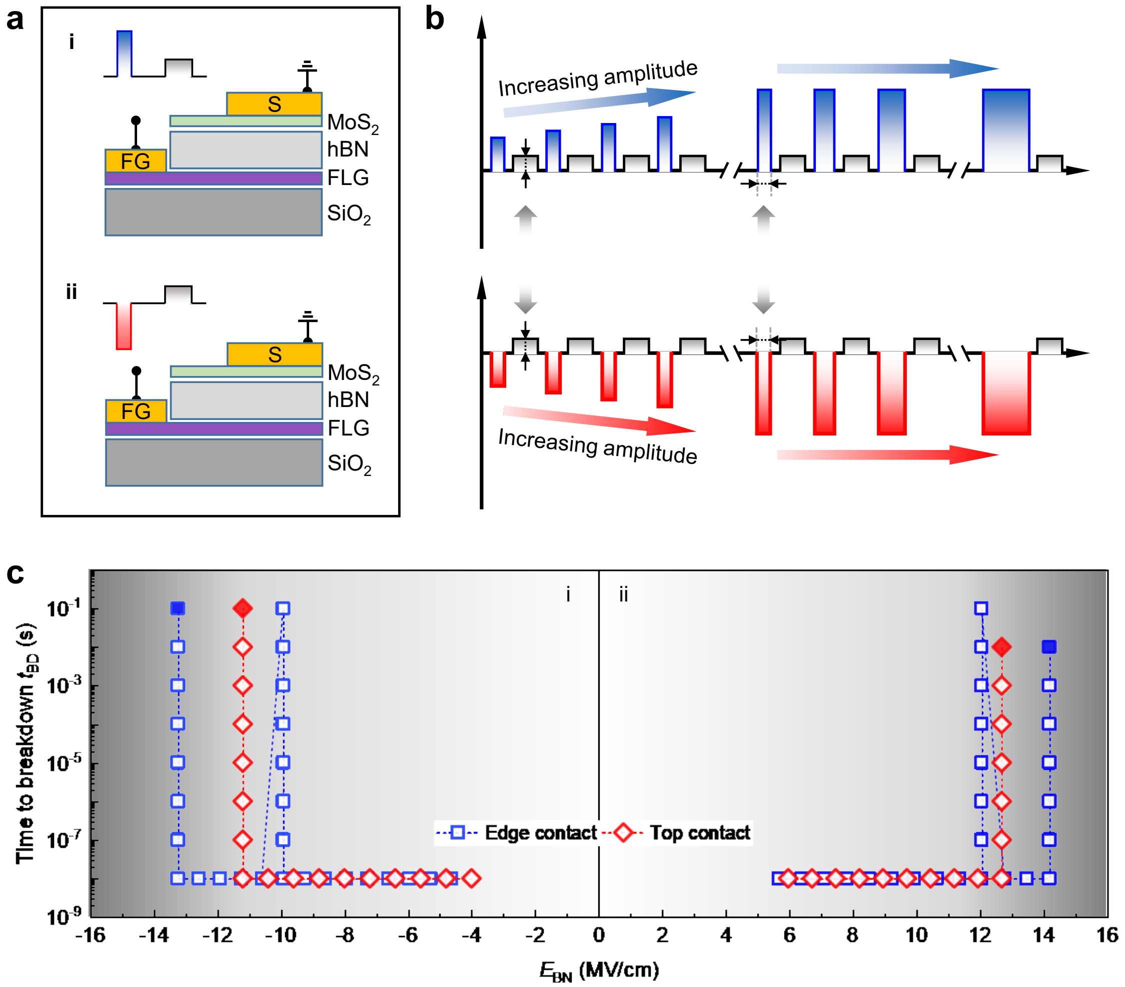

Supplementary Figure 32. Comparison of the electric field dependent time-to-breakdown for memory cells with edge contact and top contact. **a**, The detail pulse sequence of the test procedure. **b**, Schematic of the test structure and bias condition for two type pulse condition. **b**, Pulse sequence in the test for positive and negative voltage stress. **c**, The time to breakdown versus E_{BN} data for the two type contact configurations. Open marker represent that the device passes pulse stress, while the filled dot represent device breaks during the test. The hollow dot represent device could accept this pulse stress condition while the solid dot represent device is breakdown.

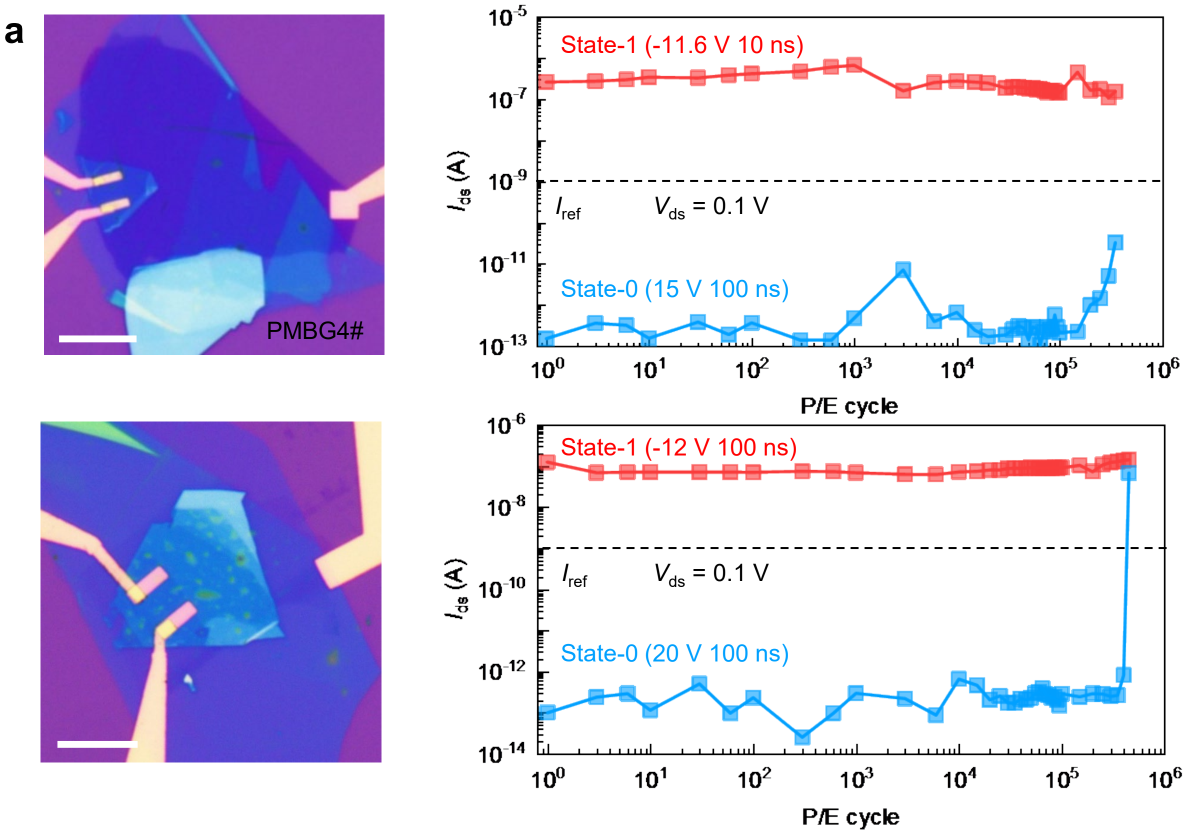

Supplementary Figure 33. Endurance performance of additional edge contacted flash memory cells (as SLC): a, PMBG4#; b, PMBG19#. The optical image and endurance characteristic are displayed for each cell. The endurance lifetime of edge contacted memory cells is ~10^5-10^6 cycles.

Supplementary Note 19. Performance comparison to the state of art flash memory cells

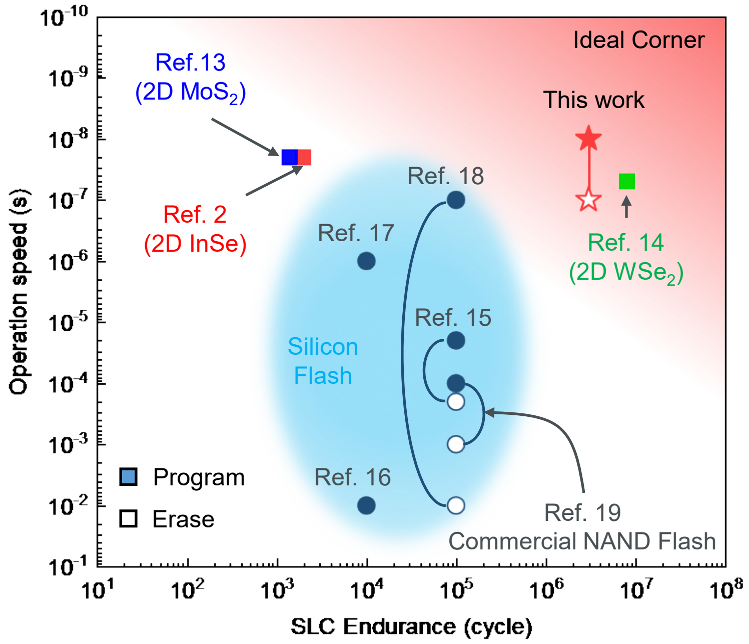

Supplementary Figure 34. Comparison of the speed-endurance characteristic of present edge contacted flash memory as SLC to previous 2D flash memory[2, 13, 14] and silicon flash memory[15-19].

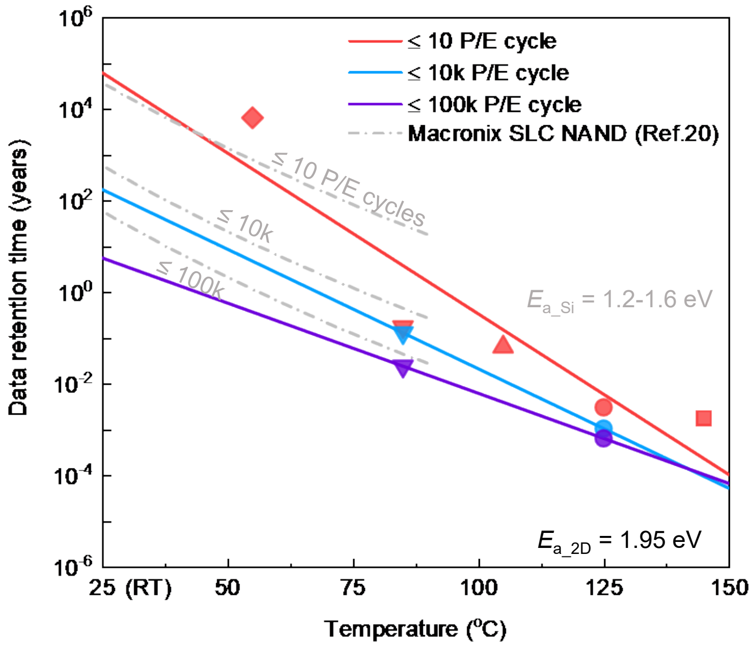

Supplementary Figure 35. The temperature dependent evaluation of retention-endurance characteristic of present edge contacted flash memory cell. After 10^5 program/erase (P/E) cycles, the memory still manifest long-term data retention over years, comparable to commercial flash memory (Macronix SLC NAND Flash)[20].

Supplementary Table 2. State of art performance of 2D flash memory compared to silicon flash.

	Functional layer	Voltage	Speed	Retention	Endurance (SLC)	Endurance (MLC)
2D flash memory	1T-2H-MoS_2/hBN/Gr (This work)	±15 V	100 ns	>10^4 s (>10 year by acceleration, 85 °C)	>10^6	> $8x10^4$
	WSe_2/hBN/HfO_2 /Al_2O_3[13]	±30 V	50 ns	>10^3 s (>10 year by acceleration, 150 °C)	>10^6	N. A.
	MoS_2/hBN/Gr[14]	±30 V	20 ns	>10^5 s (>10 year by acceleration, 85 °C)	>1390	N. A.
	InSe/hBN/Gr[2]	-20.8 V +20.2 V	21 ns	>10^3 s (>10 year by extrapolation)	>2000	N. A.
	MoS_2/hBN/Gr[21]	±15 V	100 µs	>1400 s (>10 year with extrapolation)	>100	N. A.
	MoS_2/hBN/Au[22]	±5 V	100 ms	>10^5 s (>10 year by extrapolation)	>10^5	N. A.
	MoS_2/HfO_2/Gr[23]	±15 V	100 ms	>2000 s (>10 year by extrapolation)	>120	N. A.
	BP/Al_2O_3/Al_2O_3[24]	±20 V	100 ms	>10^3 s ($2x10^7$ by extrapolation)	>100	N. A.
	MoS_2/hBN/BP[25]	±20 V	300 ms	>10^3 s	>50	N. A.
Silicon flash memory	HfO_2/Si_3N_4 stacked trapping layer Flash[15]	±15 V	20 µs 200 µs	>10^4 s (85 °C)	>10^5	N. A.
	Metal floating-gate with SiO_2/HfO_2 dual-layer tunneling barrier[16]	±12 V	10 ms 1 ms	>10^4 s (25 °C)	>10^4	N. A.
	SONNOS (Si/SiO_2/double stacked Si_3N_4)[17]	-16 V +19 V	1 µs	>10^4 s (>10 year by acceleration, 85 °C)	>10^4	N. A.
	SNOncOS (Si/SiO_2/Si_3N_4/Si-NCs/Si_3N_4)[17]	-18 V +17 V	1 µs	>10^4 s (>10 year by acceleration, 85 °C)	>10^4	N. A.
	Si_3N_4/ZrO traping layer Flash[18]	±14 V	100 ns 100 ms	>$3×10^5$ s (>10 year by acceleration, 85 °C)	>10^5	N. A.

Supplementary Table 3. State of art performance of 2D flash memory compared to commercial flash

Memory type		Program time	Erase Time	Endurance	Ref.
NAND	MPN: MT29F2G08AABWP[26]	300 μs	2 ms	10^5	26
	MPN: MT29F16G08ABACA[27]	350 μs	1.5 ms	6×10^4	27
	MPN: MT29F2G08A[28]	220 μs	500 μs	10^5	28
NOR	MPN: TE28F128J3[28]	128 μs	1 s	10^5	28
	MPN: MT25QU256ABA[29]	120 μs	150 ms	10^5	29
	MPN: N25Q256A[30]	500 μs	250 ms	10^5	30
Serial Flash Embedded Memory	MPN: M25P128[31]	500 μs	500 μs	10^5	31
2D Flash	1T-2H-MoS_2/hBN/Gr	10 ns	100 ns	3×10^6	This work

**MPN: Manufacturer Product Number*

Supplementary Note 20. Realization of 4-bit memory states

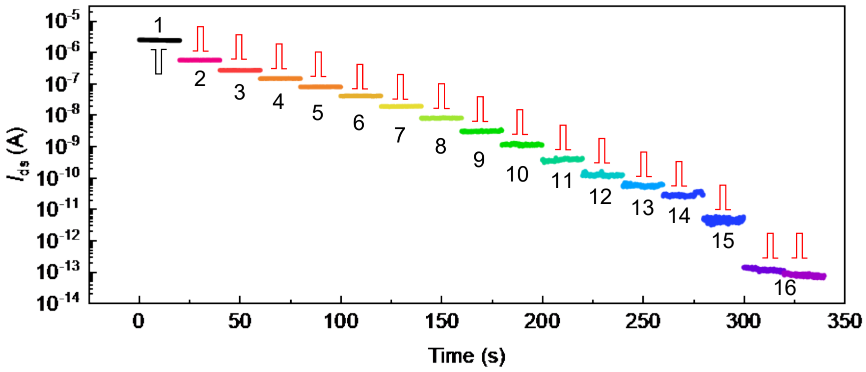

Supplementary Figure 36. The realization of Quadruple-Level Cell in the edge contacted flash memory cell (PMBG1#). The memory cell is set to the high conductive state by applying a negative V_{CG} pulse (-15 V, 10 ns). And other states are achieved by sequentially applying V_{CG} pulse of identical amplitude and width (12 V, 10 ns).

Supplementary References

1. Liu, C. et al. A semi-floating gate memory based on van der Waals heterostructures for quasi-non-volatile applications. *Nat. Nanotechnol.* **13**, 404-410 (2018).
2. Wu, L. et al. Atomically sharp interface enabled ultrahigh-speed non-volatile memory devices. *Nat. Nanotechnol.* **16**, 882-887 (2021).
3. Chen, X. et al. Probing the electron states and metal-insulator transition mechanisms in molybdenum disulphide vertical heterostructures. *Nat. Commun.* **6**, 6088 (2015).
4. Zhao, P. et al. Evaluation of border traps and interface traps in HfO_2 /MoS_2 gate stacks by capacitance–voltage analysis. *2D Mater.* **5**, 031002 (2018).
5. McCreary, K. M., Hanbicki, A. T., Sivaram, S. V., & Jonker, B. T. A- and B-exciton photoluminescence intensity ratio as a measure of sample quality for transition metal dichalcogenide monolayers. *APL Mater.* **6**, 111106 (2018).
6. Shen, T. et al. High mobility monolayer MoS_2 transistors and its charge transport behaviour under E-beam irradiation. *J. Mater. Sci.* **56**, 5307-5308 (2021).
7. Liu, L. et al. Transferred van der Waals metal electrodes for sub-1-nm MoS_2 vertical transistors. *Nat. Electron.* **4**, 342-347 (2021).
8. Liu, Y. et al. Approaching the Schottky–Mott limit in van der Waals metal–semiconductor junctions. *Nature* **557**, 696-700 (2018).
9. Das, S., Chen, H.-Y., Penumatcha, A. V., & Appenzeller, J. High performance multilayer MoS_2 transistors with scandium contacts. *Nano Lett.* **13**, 100-105 (2013).
10. Salvo, B. D. et al. Experimental and theoretical investigation of nonvolatile memory data-retention. *IEEE Trans. Electron Devices* **46**, 1518-1524 (1999).
11. Janai, M. Data retention, endurance and acceleration factors of NROM devices. In *2003 41st Annual Proceedings: International Reliability Physics Symposium (IRPS)*, 502-505 (IEEE, 2003).
12. Park, H. et al. Impact of SiO_2/Si interface micro-roughness on SILC distribution and dielectric breakdown: a comparative study with atomically flattened devices. In *2017 IEEE International Reliability Physics Symposium (IRPS)* (IEEE, 2017).
13. Huang, X. et al. An ultrafast bipolar flash memory for self-activated in-memory computing. *Nat. Nanotechnol.* **18**, 486-492 (2023).
14. Liu, L. et al. Ultrafast non-volatile flash memory based on van der Waals heterostructures. *Nat. Nanotechnol.* **16**, 874-881 (2021).
15. Chen, C., Chang-Liao, K., Wu, K., & Wang, T. Improved erasing speed in junctionless flash memory device by HfO_2/Si_3N_4 stacked trapping layer. *IEEE Electron Device Lett.* **34**, 993-995 (2013).
16. Chen, G. et al. Metal floating gate memory device with SiO_2/HfO_2 dual-layer as engineered tunneling barrier. *IEEE Electron Device Lett.* **35**, 744-746 (2014).
17. Lin, Y. et al. Comparison with nitride interface defects and nanocrystals for charge trapping layer nanowire gate-all-around nonvolatile memory performance. *IEEE Trans. Electron Devices* **65**, 493-

498 (2018).

18. Fang, H. K. et al. Operation characteristics of gate-all-around junctionless flash memory devices with Si_3N_4/ZrO-based stacked trapping layer. *IEEE Trans. Electron Devices* **67**, 3626-3631 (2020).
19. Advani, R. N. *3D Flash Memories* Ch. 1 (Springer Netherlands, 2016).
20. Macronix international Co., Ltd. *Rrogram/erase cycling endurance and data retention of macronix SLC NAND flash memories*. (2014). (Technology Note NO. AN0339V1) Retrieved from Macronix international Co., Ltd. website: https://www.macronix.com.
21. Sup Choi, M. et al. Controlled charge trapping by molybdenum disulphide and graphene in ultrathin heterostructured memory devices. *Nat. Commun.* **4**, 1624 (2013).
22. Wang, S. P. et al. New floating gate memory with excellent retention characteristics. *Adv. Electron. Mater.* **5**, 1800726 (2019).
23. Bertolazzi, S., Krasnozhon, D., & Kis, A. Nonvolatile memory cells based on MoS_2/graphene heterostructures. *ACS Nano* **7**, 3246-3252 (2013).
24. Tian, H. et al. A dynamically reconfigurable ambipolar black phosphorus memory device. *ACS Nano* **10**, 10428-10435 (2016).
25. Li, D. et al. Nonvolatile floating-gate memories based on stacked black phosphorus-boron nitride-MoS_2 heterostructures. *Adv. Funct. Mater.* **25**, 7360-7365 (2015).
26. Micron Technology, Inc. *2, 4, 8Gb: x8/x16 Multiplexed NAND Flash Memory Features*. (2004). (Data Sheet NO. 09005aef81590bdd) Retrieved from Micron Technology, Inc. website: https://www.micron.com/.
27. Micron Technology, Inc. *16Gb, 32Gb, 64Gb Asynchronous/Synchronous NAND Features*. (2010). (Data Sheet NO. 09005aef844588dc) Retrieved from Micron Technology, Inc. website: https://www.micron.com/.
28. Micron Technology, Inc. *NAND Flash 101 Introduction*. (2006). (Technical Note NO. TN-29-19) Retrieved from Micron Technology, Inc. website: https://www.micron.com/.
29. Micron Technology, Inc. *256Mb, 1.8V Multiple I/O Serial Flash Memory Features*. (2014). (Data Sheet NO. CCMTD-1725822587-3458) Retrieved from Micron Technology, Inc. website: https://www.micron.com/.
30. Micron Technology, Inc. *3V, 256Mb: Multiple I/O Serial Flash Memory Features*. (2011). (Data Sheet NO. 09005aef84566603) Retrieved from Micron Technology, Inc. website: https://www.micron.com/.
31. Micron Technology, Inc. *M25P128 Serial Flash Embedded Memory Features*. (2016). (Data Sheet NO. CCMTD-1718347970-10412) Retrieved from Micron Technology, Inc. website: https://www.micron.com/.
